# Immunophenotype profile by flow cytometry reveals different subtypes of extracellular vesicles in porcine seminal plasma

**DOI:** 10.1186/s12964-024-01485-1

**Published:** 2024-01-23

**Authors:** Isabel Barranco, Alberto Alvarez-Barrientos, Ana Parra, Pablo Martínez-Díaz, Xiomara Lucas, Jordi Roca

**Affiliations:** 1 Department of Medicine and Animal Surgery, Faculty of Veterinary Science, University of Murcia, Murcia, Spain; 2 Servicio de Técnicas Aplicadas a las Biociencias, Universidad de Extremadura, Badajoz, Spain

**Keywords:** Ejaculate, Extracellular vesicles, Flow cytometry, Pig, Seminal plasma

## Abstract

**Background:**

Porcine seminal plasma (SP) is endowed with a heterogeneous population of extracellular vesicles (sEVs). This study evaluated the immunophenotypic profile by high-sensitivity flow cytometry of eight sEV subpopulations isolated according to their size (small [S-sEVs] and large [L-sEVs]) from four different SP sources, namely three ejaculate fractions (the first 10 mL of the sperm rich fraction [SRF-P1], the remaining SRF [SRF-P2], and the post-SRF [PSRF]) and entire ejaculate (EE).

**Methods:**

Seminal EVs were isolated using a size exclusion chromatography-based protocol from six SP pools (five ejaculates/pool) of each SP source and characterized using complementary approaches including total protein (BCA™assay), particle size distribution (dynamic light scattering), morphology (transmission electron microscopy), and purity (albumin by Western blot). Expression of CD9, CD63, CD81, CD44 and HSP90β was analyzed in all sEV subpopulations by high-sensitivity flow cytometry according to MIFlowCyt-EV guidelines, including an accurate calibration, controls, and discrimination by CFSE-labelling.

**Results:**

Each sEV subpopulation exhibited a specific immunophenotypic profile. The percentage of sEVs positive for CD9, CD63, CD81 and HSP90β differed between S- and L-sEVs (*P* < 0.0001). Specifically, the percentage of sEVs positive for CD9 and CD63 was higher and that for CD81 was lower in S- than L-sEVs in the four SP sources. However, the percentage of HSP90β-positive sEVs was lower in S-sEVs than L-sEVs in the SRF-P1 and EE samples. The percentage of sEVs positive for CD9, CD63, and CD44 also differed among the four SP sources (*P* < 0.0001), being highest in PSRF samples. Notably, virtually all sEV subpopulations expressed CD44 (range: 88.04–98.50%).

**Conclusions:**

This study demonstrated the utility of high-sensitivity flow cytometry for sEV immunophenotyping, allowing the identification of distinct sEV subpopulations that may have different cellular origin, cargo, functions, and target cells.

**Supplementary Information:**

The online version contains supplementary material available at 10.1186/s12964-024-01485-1.

## Background

Extracellular vesicles (EVs) are membrane-surrounded particles of 30–1000 nm that carry proteins, lipids, metabolites, and nucleic acids and are released into the extracellular environment by most body cells [1]. EVs circulate freely in all body fluids and represent an important cell-to-cell communication pathway that allows donor cells to exchange molecular messages with nearby or distant target cells, eliciting a specific response in the latter [2]. In recent years, EVs have become attractive targets for the scientific community due to their potential utility as therapeutic agents and as biomarkers for the diagnosis/prognosis of a wide range of body pathologies, including cancer [2, 3] and fertility disorders [4, 5].

Despite the great scientific relevance of EVs, many fundamental insights into their phenotypic characteristics and biological functions remain to be elucidated. The EV population present in any biological sample is heterogeneous, with several subpopulations coexisting that differ in phenotypic characteristics, such as size, electron density or morphology, and also in molecular composition. These differences define the functional role of EVs in target cells and are determined by the cellular source and the biogenesis mechanism [6, 7]. Consequently, one of the major challenges facing EV researchers is to discern this diversity of EVs in any biological sample. This is an essential step to better understand the functional role of EVs in both physiological and pathological processes, but a task that is hampered by the lack of reliable and accurate methods to separately characterize each EV subpopulation separately [8, 9]. To overcome this shortcoming, current characterization methods, which are based on mass measurements that provide average data over the entire EV population, need to be replaced by high-throughput methods that allow reliable characterization of individual EVs [10].

Flow cytometry would be one of the high-throughput technologies suitable for the characterization of individual EVs [11, 12]. Indeed, flow cytometry, a laser-based technology, has proven to be an efficient tool for the multiparametric analysis of single cells and particles [13]. Technical advances in recent years have enabled the development of highly sensitive flow cytometers optimized for nanoparticle analysis [14]. Flow cytometers that can also discriminate between EVs and non-EV particles, providing accurate information on the number, size, and molecular phenotype (using fluorescent probes) of large numbers of individual EVs [15].

EVs have been isolated from several reproductive fluids, including seminal plasma (SP), a complex fluid secreted primarily by the male accessory sex glands [16, 17]. The SP contains a large and heterogeneous population of EVs (sEVs) that would be involved in modulating sperm physiology and functionality, including epididymal maturation, motility, capacitation, and acrosome reaction, as well as in regulating the immune environment of the female genital tract [16]. Despite this functional relevance, sEVs remain comparatively less studied than those circulating in other body fluids [18, 19]. Based on these arguments, the aim of this study was to characterize by flow cytometry the immunophenotypic profile of eight subpopulations of sEVs, namely two populations of different size (large and small EVs [L-sEVs and S-sEVs, respectively]) isolated from four different SP sources (from three ejaculate fractions and from entire ejaculates). Ejaculates were obtained from pigs, an animal with fractionated ejaculates like humans and considered a suitable animal model for human reproductive medicine [20, 21]. Immunophenotyping of sEVs by flow cytometry was performed according to the Minimum Information Framework for flow cytometry experiments (MIFlowCyt-EV [22]) using a CytoFLEX S, a high-sensitivity flow cytometer that has been shown to be effective for EV detection [13, 23], together with a combination of fluorescent antibodies against EV-proteins.

## Methods

### Ethical statement

All procedures involving animals were conducted in accordance with the European guidelines for the protection of animals used in scientific research (Directive 2010–63-EU) and approved by the Bioethics Committee of University of Murcia (Murcia, Spain; CBE: 367/2020 and CBE: 538/2023). The boar ejaculates were provided by a commercial artificial insemination (AI) center (AIM Iberica, Calasparra, Murcia, Spain). The center complies with the Spanish (ES300130640127, August 2006) and European (ES13RS04P, July 2012) guidelines for the production and marketing of AI semen doses and animal health and welfare.

### Animals, ejaculates, and seminal plasma collection

Semen samples were collected from healthy, sexually mature (18 to 36 months) and fertile Landrace and Large White boars used in commercial AI programs and housed in individual pens within an environmentally controlled building (15–25 °C and 16 h/day of natural and supplemental light). The AI boars had* ad libitum* access to water and were fed commercial diets designed to meet the nutritional requirements of AI boars.

Ejaculates (*n* = 30) were collected by the gloved hand method in three separate fractions, namely, the first 10 mL of the sperm rich ejaculate fraction (SRF-P1), the remaining SRF (SRF-P2), and the post-SRF (PSRF). A proportional aliquot of each ejaculate fraction was mixed to reconstitute that mimicked the entire ejaculate (EE). Thus, for each of the 30 ejaculates collected, there were four separate semen samples, the three fractions and the entire ejaculate, for a total of 120 semen samples. All the ejaculates included in this study met the sperm quality threshold requirements to produce commercial semen AI doses, namely, sperm concentration ≥ 200 × 10^6^ sperm/mL, total sperm motility ≥70% and normal sperm morphology ≥75%.

Immediately after ejaculate collection, each semen sample was centrifuged twice at 1500 xg for 10 min at room temperature (RT) (Rotofix 32A, Hettich Centrifuge UK, Newport Pagnell, Buckinghamshire, England, UK) and the resulting supernatant (i.e. SP) was examined microscopically (Eclipse E400; Nikon, Tokyo, Japan) to ensure that no spermatozoa or other cellular debris remained. The SP samples were then treated with a protease inhibitor cocktail (Roche complete™ Protease Inhibitor Cocktail tablets; Basilea, Switzerland) and stored at 5 °C (Zanussi Tropic System, Electrolux España S.A.U., Madrid, Spain) until sEV isolation (less than 24 h after ejaculate collection).

### Isolation of seminal extracellular vesicles

**Fig. 1:**
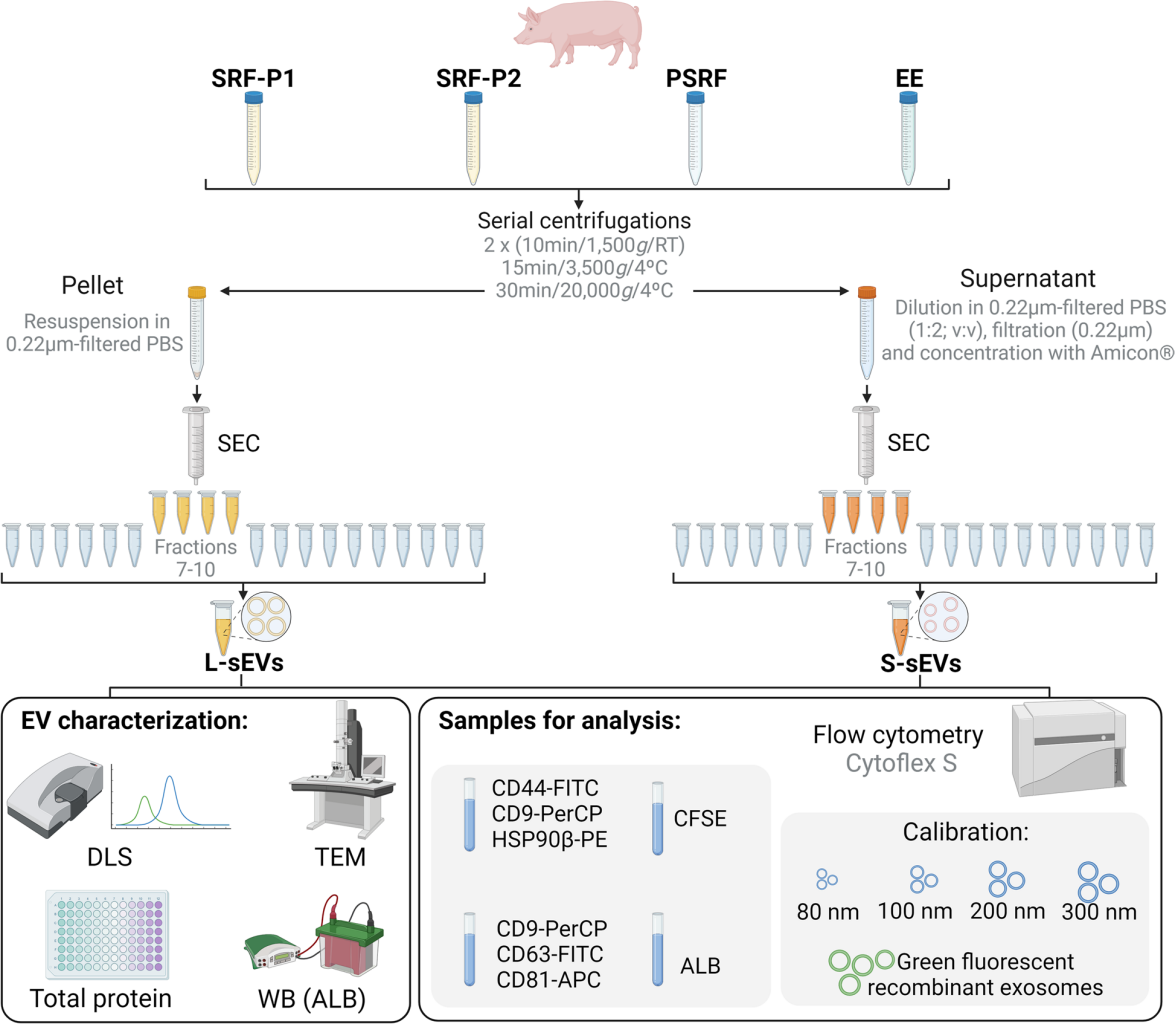
Flowchart of study design. ALB: albumin; APC: allophycocyanin; DLS: dynamic light scattering; EV: extracellular vesicles; EE: entire ejaculate; FITC: Fluorescein isothiocyanate; L-sEVs: large seminal EVs; PE: Phycoerythrin; PerCP: Peridinin Chlorophyll Protein; PSRF: Post sperm rich ejaculate fraction; S-sEVs: small seminal EVs; SEC: size exclusion chromatography; SRF-P1: the first 10 mL of the sperm rich ejaculate fraction; SRF-P2: the remaining sperm rich ejaculate fraction; TEM: transmission electron microscopy; WB: Western blot

Seminal EVs were isolated using a size exclusion chromatography (SEC)-based method [24]. First, the 30 SP samples from each of the four SP sources (SRF-P1, SRF-P2, PSRF, and EE) were randomly pooled to create 6 SP pools (5 SP samples for each SP source), totaling 24 SP samples (Fig. 1). The resulting SP samples (6 mL-aliquot each) were then centrifuged at 3200 x*g* for 15 min at 4 °C (Sorvall™ STR40, Thermo Fisher Scientific, Waltham, MA, USA) to ensure the absence of cells or debris. The supernatants (4 mL) were then centrifuged at 20,000 x*g* for 30 min at 4 °C (Sorvall™ Legend™ Micro 21R, Thermo Fisher Scientific), and the resulting pellets and supernatants were processed separately for SEC. The pellets were diluted in 500 μL of 0.22 μm filtered phosphate buffered saline (PBS, Merck^®^, Darmstadt, Germany). The supernatants (2 mL) were diluted in 0.22 μm filtered PBS (1:2; v:v), filtered at 0.22 μm (Millex^®^ Syringe Filters, Merck^®^), and concentrated by three repeated centrifugations at 3200 x*g* for 30 min at 4 °C using Amicon® Ultra-4 mL centrifugal filter 100 kDa (Merck^®^) until a volume of 2 mL. SEC columns were homemade using filtration tubes (Econo-Pac^®^ Chromatography Columns, Bio-Rad, Hercules, California, USA) and Sepharose CL2B^®^ (10 mL, Merck^®^). Briefly, the filtration tubes were placed on a retort stand and Sepharose CL2B^®^ was added dropwise, alternating with the same volume of 0.22 μm filtered PBS. The Sepharose CL2B^®^ was then allowed to settle to the bottom of the filtration tube until 10 mL was reached. Finally, the SEC columns were equilibrated and washed between runs with 0.22 μm filtered PBS (60 mL). The two samples (i.e., pellet [500 μL] and supernatant [2 mL] of 20,000 x*g*) of each SP sample were applied separately to the SEC column. Twenty 500 μL eluting fractions were separately collected from each sample and the sEV-enriched fractions (7 to 10) were selected and mixed. These fractions were selected based on the results of our previous experiments (unpublished data), which showed that they were the most enriched in sEVs. Forty-eight sEV samples were generated (24 from 20,000 x*g* pellets [enriched in large sEV subset, L-sEVs], and 24 from 20,000 x*g* supernatants [enriched in small sEV subset, S-sEVs]). Finally, each sEV sample was aliquoted into two separate cryovials and stored at − 80 °C (Ultra Low Freezer; Haier Inc., Qingdao, China). One cryovial was used to characterize sEV and the other to immunophenotype sEV using high-sensitivity flow cytometry (Fig. 1).

### Seminal extracellular vesicles characterization

The isolated sEVs were characterized according to the Minimal information for studies of extracellular vesicles recommendations (MISEV 2018) [25] using multiple and complementary approaches: (1) concentration of sEVs measured indirectly by measuring the total protein concentration, (2) particle size distribution by dynamic light scattering (DLS), (3) morphology of sEVs by transmission electron microscopy (TEM), and (4) purity of sEVs by measuring the albumin content by Western blot (WB).

Total protein concentration was measured using bicinchoninic acid assay (BCA) following manufacturer guidelines (Thermo Fisher Scientific). Prior to analysis, sEVs were lysed by mixing 25 μL of sEV samples with of 25 μL of lysis solution (Triton [0.1%, Merck^®^] and sodium dodecyl sulfate [SDS, 0.1%, Merck^®^]) and incubated at 37 °C with agitation (50 rpm) for 30 min. Absorbance was read in a microplate reader (wavelength: 570 nm; PowerWave XS; Bio-Tek Instruments, Winooski, Vermont, USA).

Particle size distribution and Zeta potential were analyzed by DLS using a Zetasizer Nano ZS system (Malvern Panalytical, Malvern, UK) recording backscattered light at 173° and operating at 633 nm at RT. Briefly, sEV samples were shaken (50 rpm for 30 s) to avoid sEV aggregation and placed in 10 mm pathlength cuvettes (50 μL for particle size distribution) or disposable folded capillary cuvettes (750 μL for Zeta potential). Light scattering was measured for 150 s and one (Zeta potential) or three (particle size distribution) assessments were performed per sample. The setup used for the DLS measurement was as follows: Refractive index *n* = 1.33; System temperature: 25 °C; Sample equilibration: 60 s; Absorption k = 0.01. Data were obtained using Dispersion Technology Software v.5.10 (Malvern Panalytical). The particle size distribution results were plotted as intensity and expressed as a range (25-75^th^ percentiles).

The morphology of sEVs was analyzed by TEM following the protocol described by Thery et al. [26] with minor modifications. Briefly, 5 μL of sEV samples were fixed in 1 μL of 0.1% paraformaldehyde (Merck^®^) and placed on carbon-coated formvar electron microscopy grids for 7 min at RT. The grids were fixed in 10 μL of 2% uranyl acetate and examined using a JEOL JEM 1011 TEM (JEOL Ltd., Tokyo, Japan). Images were captured with an Orius SC200 camera (Gatan, Evry, France).

The purity of the sEV samples was assessed by WB evaluating the presence of albumin, a protein abundant in porcine SP. Briefly, SP (positive control) and sEV samples were denatured at 95 °C for 5 min and loaded onto a 4–12% SDS-PAGE. Electrophoresis was performed at 180 V for 60 min and proteins were transferred to a Polyvinylidene Difluoride membrane (Amersham Hybond P 0.45 PVDF, GE Healthcare Life Sciences, Chicago, Illinois, USA) by semidry electrophoresis at 1.5 mm, 25 V, 1.3A program (Invitrogen from Power Blotter Station, model: PB0010, Thermo Fisher Scientific). The membranes were blocked for 45 min with Tris-buffered saline (TBS)-Roti^®^-Block × 10 (CARL ROTH, Karlsruhe, Baden-Württemberg, Germany) and incubated overnight at 4 °C with rabbit polyclonal anti-porcine albumin antibody (1:1000, CLFAG16140, Burlington, Ontario, Canada). The next morning and after three washes with TBS plus 0.1% Tween 20 (Merck^®^, 5 min each), the membranes were incubated with a horseradish peroxidase-conjugated goat polyclonal anti-rabbit antibody (1:10000 ab6721, Abcam, Cambridge, UK) for 60 min. Immunoreactive bands were detected with ECL select WB detection reagent (Cytiva, Thermo Fisher Scientific). Images were acquired with ImageQuant LAS 500 (GE Healthcare, Chicago, Illinois, USA).

Data from the experiments were submitted to the EV-TRACK knowledgebase (EV-TRACK ID: EV231005).

### Flow cytometry immunophenotypic analysis

Immunophenotyping of sEVs was evaluated using a CytoFLEX S flow cytometer (Beckman Coulter, Life Sciences Division Headquarters, Indianapolis, USA). The flow cytometer was equipped with four lasers, namely violet, blue, yellow, and red (405 nm, 488 nm, 561 nm, and 638 nm, respectively), to detect up to 13 fluorescence parameters. A detailed description of the flow cytometry analysis, including pre-analytical and analytical procedures, is provided in the MIFlowCyt-EV reports in the Additional files 1 and 2.

#### Calibration and setup of extracellular vesicles detection region

First, the optical setup of the flow cytometer was modified to use the side scatter (SSC) information of the 405 nm laser (violet-SSC) instead of the 488 nm laser. The SSC was then calibrated using polystyrene beads of known diameter between 80 and 300 nm with a density of 1,05 g/cm^3^ and a refractive index of 1.59 nm (Cat 30080A, 30100A, 30200A and 30300A, Nanosphere™ serie 3000; Thermofisher Scientific, Waltham, Massachusetts, USA). The forward scatter (FSC) and violet-SSC parameters were corrected on a logarithmic scale and the fluorescence channels were corrected on a logarithmic gain. The EV detection gate was then set using dot plot of FSC-H vs violet-SSC-H parameters. The SSC data generated by beads were fitted to nm according to Mie theory, using FCMPASS software (https://nano.ccr.cancer.gov/fcmpass/). The lower limit of the flow cytometer detection was 80 nm. This is equivalent to a mean of 145 nm (range of 118 to 165 nm) in the FCMPASS software for EVs. These data were consistent with DLS particle size distribution measurements. Commercially available recombinant exosomes expressing green fluorescent protein (GFP) on their membrane surface (SAE0193, Merck^®^) with a size distribution ranging from 30 to 200 nm (peak at 100–150 nm, measured by DLS) were used to validate the accuracy of the flow cytometer for the analysis of sEVs. The concentration of the recombinant fluorescent exosomes used was 1 × 10^6^ (the actual concentration range between 0.85 × 10^6^ and 0.93 × 10^6^, depending on the batch). It was within the expected concentration range of sEVs from the samples to be analyzed. The flow cytometer was subjected to a quality check on each working day, which included a test with recombinant exosomes expressing GFP.

#### Labeling of seminal extracellular vesicles prior to immunophenotyping analysis

Prior to immunophenotyping analysis, sEV samples were incubated with CellTrace™ carboxyfluorescein succinimidyl ester (CFSE, Thermo Fisher Scientific), a non-fluorescent probe that becomes fluorescent upon contact with active esterases present only in functional intact membrane structures [27]. CFSE-labeling was used to distinguish intact and functional sEVs from other non-EV particles and membrane fragments. The CFSE stock solution (10 μM) was prepared by adding 90 μL of dimethyl sulfoxide (DMSO) to the vial of CFSE powder. The working solution was prepared by diluting 5 μL of the CFSE stock solution in 495 μL of 0.1 μm filtered PBS (1:100) after titration. The mixture was centrifuged three times at 17,000 x*g* for 10 min and the supernatant was used for incubation with sEVs. Ten μL of each sEVs sample was incubated with 100 μL of CFSE working solution for 30 min at 37 °C in the dark. CFSE-positive events within the EV detection region were considered sEVs. Detergent-treated sEV samples (0.1% Triton and 0.1% SDS in 0.22 μm filtered PBS) were used as a negative control (lysed sEV samples) to verify that only intact sEVs were CSFE positive.

#### Antibodies and preparation of working solutions

For the immunophenotyping of EVs, some of the proteins recommended by the MISEV2018 guidelines for EV characterization were analyzed based on their protein content [25]. The proteins analyzed were the tetraspanins CD9, CD63 and CD81 and the cytosolic protein HSP90β, which has the ability to bind membrane proteins. In addition, the transmembrane protein CD44, known to be present in porcine sEVs [28], was also analyzed. Table 1 shows the details of the fluorescence-conjugated antibodies used. The antibodies against the tetraspanins CD9, CD63 and CD81 react with human target proteins. For use in the present experiment, amino acid sequence alignment between human and porcine was checked using the EMBOSS Matcher software (https://www.ebi.ac.uk/Tools/psa/emboss_matcher/). The check revealed 89, 86.6, and 95.8% identity and 95.2, 91.2, and 98.3% similarity for CD9, CD63, and CD81, respectively. These results strongly suggest that these antibodies can correctly identify the three porcine tetraspanins. Albumin was also analyzed as a non-EV protein to verify the purity of the sEV samples (Note that the presence of albumin was also analyzed by WB). Working solutions for each antibody were prepared by diluting the antibodies in 0.1 μm filtered PBS and following manufacturer recommendations: 1:50 for anti-CD9, anti-CD63 and anti-CD81, 1:100 for anti-HSP90β, 1:75 for anti-CD44 and 1:30 for anti-albumin. The working solutions were centrifuged at 800 x*g* for 3 min using Ultrafree-MC centrifuge filters (C78142, Merck®) and the filtrates were used for immunophenotyping of sEVs. Detergent-treated sEV samples (0.1% Triton and 0.1% SDS in 0.22 μm filtered PBS) were used as control (lysed sEV samples) to verify that only intact sEVs were antibodies positive.

**Table 1:** Fluorescent conjugated antibodies used in the study

Feature	EV Protein					
	CD9	CD63	CD81	HSP90β	CD44	Albumin
**Antibody**	CD9 Antibody, anti-human	CD63 Antibody, anti-human	CD81 Antibody, anti-human	HSP90β polyclonal antibody	Rat anti mouse CD44	Anti-swine Albumin
**Conjugate**	PerCP	FITC	APC	PE	FITC	FITC
**Species reactivity**	Human	Human	Human	Pig and other	Mouse	Pig
**Pig cross-reactivity**	No^1^	No^1^	No^1^	Yes	Yes	Yes
**Manufacturer**	Miltenyi Biotec	Miltenyi Biotec	Miltenyi Biotec	Enzo life Sciences	Bio-Rad	Cedarlane Laboratories
**Code**	130–118-814	130–123-673	130–119-787	ADI-SPA-844PE	MCA4703F	CLFAG16140

Bio-Rad, Hercules, California, USA; Cedarlane Laboratories, Burlington, Canada; Enzo Life Sciences, Farmingdale, NY, USA; Miltenyi Biotec B.V. & Co. KG, Bergisch Gladbach, Germany

*EV* extracellular vesicle, *APC* allophycocyanin, *FITC* Fluorescein isothiocyanate, *PE* Phycoerythrin, *PerCP* Peridinin Chlorophyll Protein

^1^Identity and similarity of amino acid sequence alignments between humans and pigs greater than 86% (see text)

#### Immunophenotyping of seminal extracellular vesicles

The sEVs were immunophenotyped using the following antibody combinations: (1) CD9-PerCP + CD63-FITC + CD81-APC, and (2) CD44-FITC + HSP90β-PE + CD9-PerCP (Fig. 1). To define the most optimal incubation protocols, control titrations were performed for each antibody to determine the optimal concentration to best discriminate between sEVs and background. Accordingly, 10 μL of sEV samples were incubated with (1) 1 μl anti-CD9-PerCP, 1 μL anti-CD63-FITC, 1 μL anti-CD81-APC; and with (2) 2 μL anti-CD44-FITC, 1 μL anti-HSP90β-PE and 1 μL anti-CD9-PerCP. Incubations were performed for 30 min at 37 °C in the dark. Samples were resuspended in 0.1 μm-filtered PBS to a final volume of 500 μL prior to flow cytometry analysis.

#### Controls

Controls were performed according to the recommended MIFlowCyt-EV guidelines [22] to ensure the absence of background noise, autofluorescence, and non-specific antibody signal. These controls include: (1) buffer only (0.1 μm filtered PBS); (2) buffer with each fluorochrome separately; (3) unstained sEV samples; (4) single-stained sEV samples (i.e., sEV samples separately incubated with anti-CD9-PerCP, anti-CD63-FITC, anti-CD81-APC, anti-HSP90β-PE, or anti-CD44-FITC) prepared under the same incubation conditions and acquired at the same settings in Cytoflex S as the sEV-stained samples; and (5) lysed sEV samples incubated with anti-CD44-FITC + HSP90β-PE or anti-CD81-APC + CD44-FITC. These controls were able to correctly distinguish sEVs from contaminants and debris.

#### Flow cytometry acquisition settings

A wash step with 0.1 μm filtered distilled water was performed prior to sample acquisition to minimize background noise. Acquisition of sEV samples was performed when the number of events per second of 0.1 μm filtered distilled water was between 5 and 10 at a low flow rate of 10 μL/min. The analysis setup was adjusted to acquire 10 × 10^3^ events per sample. In some samples where this was not possible due to dilution rate and/or low total number of sEVs, the percentage of positive events was compared to the total number of events to avoid potential bias. To avoid swarm effect, sEV samples were diluted to ensure that no more than 120 events per second were acquired at the minimum cytometer speed (10 uL/min). Two technical replicates were analyzed for each sEV sample (coefficient of variation < 10%). Filtered distilled water (0.1 μm) was used as the sheath fluid and 0.1 μm-filtered PBS was used to identify background signals. Two-minute wash steps with 0.1 μm-filtered distilled water were performed between sEV samples. Considering the technical characteristics of Cytoflex S, PE was excited with 561 nm laser and fluorescence was collected in channel Y1 (575/25 nm filter), FITC was excited with 488 nm laser and fluorescence was collected in channel B1 (525/25-nm filter), PerCP-A was excited with 488 nm laser and fluorescence was collected in channel B2 (710/30 nm filter), and APC was excited with 633 nm laser and fluorescence was collected in channel R1 (670/40 nm filter). With this approach no compensation was needed. The SSC was obtained with a 405 nm laser and therefore the parameter was named violet SSC (vSSC). Data analysis was performed using CytoExpert software (BeckmanCoulter). Flow cytometry files were uploaded to the FlowRepository database (ID: FR-FCM-Z732).

### Statistical analysis

Data were statistically analyzed using GraphPad Prism 9.3.0 (GraphPad Software, Inc., La Jolla, CA, USA; https://www.graphpad.com/). First, the Shapiro-Wilk test was used to analyze the data for normal distribution. A two-way ANOVA analysis was then performed to analyze the influence of sEV subsets (L-sEVs and S-sEVs) and SP sources (SRF-P1, SRF-P2, PSRF, and EE) on sEV characterization parameters and on immunophenotyping of the sEV subpopulations identified in the different SP sources. Tukey’s test was used for multiple comparisons, with *P* < 0.05 accepted as the minimum significance level.

## Results

### Characterization of seminal extracellular vesicle subpopulations

The characterization of sEVs from the eight samples generated from two subsets of sEVs (L-sEVs and S-sEVs) isolated from four different SP sources (SRF-P1, SRF-P2, PSRF, and EE) is summarized in Additional files 3 and 4. Total protein concentration (μg/mL) differed between SP sources (*P <* 0.001), but not between sEV subsets, with an interaction between the two main factors (*P <* 0.001) (Additional file 3). DLS measurements revealed differences in particle size distribution between sEV subsets (*P <* 0.0001) and between SP sources (*P <* 0.0001), with no significant interaction between the main factors (Additional file 3). The particle size distribution ranged between 90 and 185 nm (25^th^ and 75^th^ percentiles, respectively) in S-sEVs and between 170 and 300 nm in L-sEVs samples. Zeta potential did not differ between the two subsets of sEVs but did differ between SP sources (*P* < 0.05), with no significant interaction between the two main factors (Additional file 3). The conductivity ranged from 13.5 to 16.8 mS/cm, values expected for samples diluted in PBS. TEM images confirmed the presence of membrane-enclosed nanostructures and showed an enrichment of small and large sEVs in S- and L-sEVs samples, respectively (Additional file 4). No relevant differences in the morphology of sEVs were observed among the eight sEV samples. WB analysis showed no presence of albumin in the eight sEV samples, confirming the high purity of sEVs in all samples (Additional file 5). In contrast to WB, flow cytometry was able to measure albumin in both subsets of sEVs, although the percentages of albumin-positive particles were low, namely 6.86% ± 3.38% (mean ± SD) in S-sEVs samples and 2.16% ± 1.90% in L-sEVs samples.

### Flow cytometry controls to identify and immunophenotype porcine seminal extracellular vesicles

The EV detection region for analysis (vSSC/FSC) was established by using recombinant exosomes expressing GFP (control EVs). The fluorescence of GFP was used as the threshold (the fluorescence threshold was set to 800). The threshold was adjusted after defining and identifying the region where more than 90% of the events were displayed on the vSSC/FSC dot plot (vSSC:104826 and FSC:1000). The region was defined as the EV detection region (Additional file 6). For sEVs analysis, vSSC and FSC were used as thresholds and all events within the EV detection region were considered. This ensures that all EVs are analyzed regardless of fluorescent staining. The SSC dot plot in Additional file 6 shows the distribution of the control EVs, noting that most of them were within the EV detection region. In addition, CFSE staining was performed on each sEV sample to confirm the vSSC/FSC boundaries and the quality of the sEV sample preparation (Additional file 6). Samples of lysed sEVs (treated with the combination of Triton [0.1%] and SDS [0.1%] detergents in 0.22 μm filtered PBS; 1:1, v:v) incubated with CFSE were loaded and gated as a negative control. As expected, no CFSE positive events were detected. Subsequently, fluorescence controls were performed to ensure the staining specificity of the CD9, CD63, CD81, CD44 and HSP90β antibodies (Additional files 7, 8 and 9). The controls showed that the five antibodies tested bound adequately to the sEVs. Each antibody showed measurable levels of fluorescence at the chosen concentration (Additional file 8). The controls also demonstrated that the antibodies bound to intact sEVs (CSFE positive events), as they exhibited no fluorescence when the stained samples contained lysed sEVs (Additional file 9).

### Immunophenotyping of seminal extracellular vesicle subpopulations from the different seminal plasma sources

**Fig. 2:**
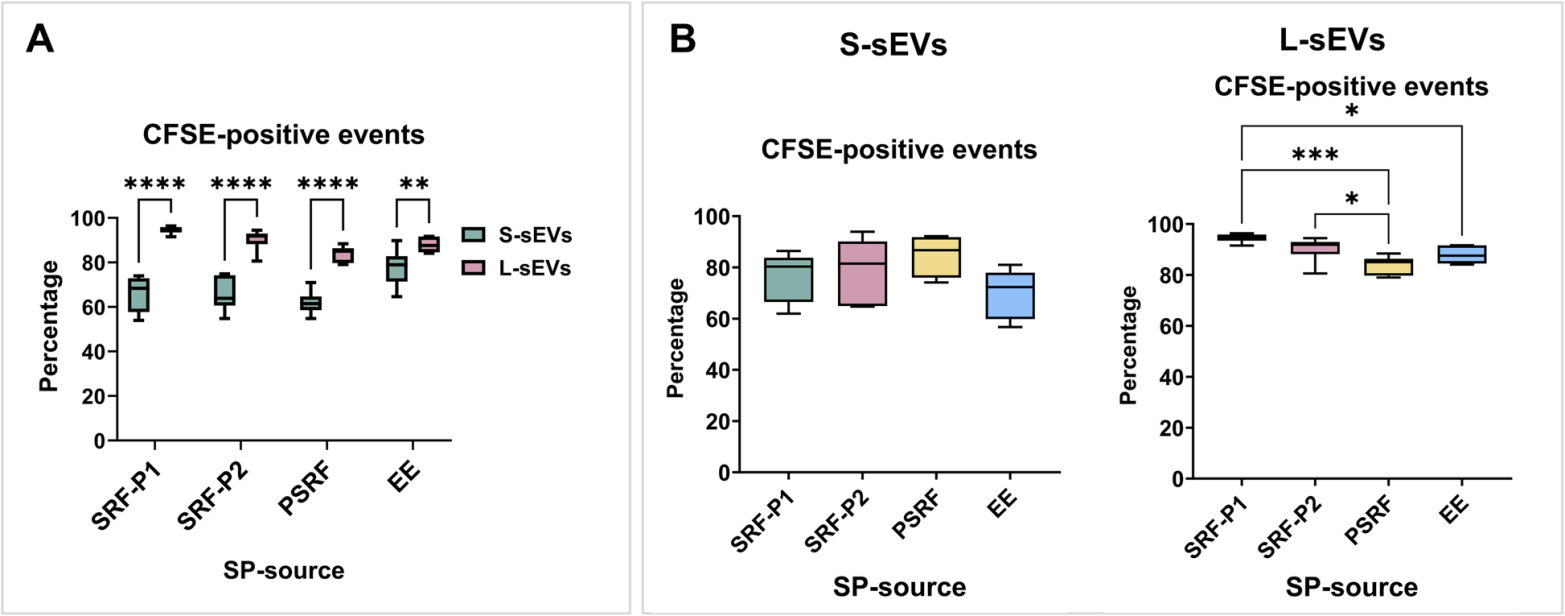
Box plots showing the percentages of carboxyfluorescein succinimidyl ester (CFSE)-positive events in samples of extracellular vesicles (EVs) isolated from seminal plasma (SP) of porcine ejaculates (sEVs). Seminal EVs were isolated by SEC in two different size subsets (small [S-sEVs] and large [L-sEVs]) and from four different SP sources: the first 10 mL of the sperm rich ejaculate fraction (SRF-P1), the remaining of the SRF (SRF-P2), the post-SRF (PSRF), and from the entire ejaculate (EE). Data from six replicates. Boxes enclose the 25^th^ and 75^th^ percentiles, whiskers extend to the 5^th^ and 95^th^ percentiles, and the line indicates the median. Different asterisks indicate different statistical values (*: *P* < 0.05; **: *P* < 0.01; ***: *P* < 0.001; ****: *P* < 0.0001)

The percentage of CFSE-positive events ranged from 53.99 to 96.39% and differed between the two sEV subsets (*P <* 0.0001) and the four SP sources (*P < *0.01), with a significant interaction (*P <* 0.01) between them (Fig. 2). The percentage of CFSE-positive events was lower in S- than in L-sEVs for all SP sources (Fig. 2A). In the L-sEVs, the percentage was higher in SRF samples (P1 and P2) than in PSRF and EE samples. In the S-sEVs, no differences were found between SP sources in the percentage of CFSE-positive events.

**Fig. 3:**
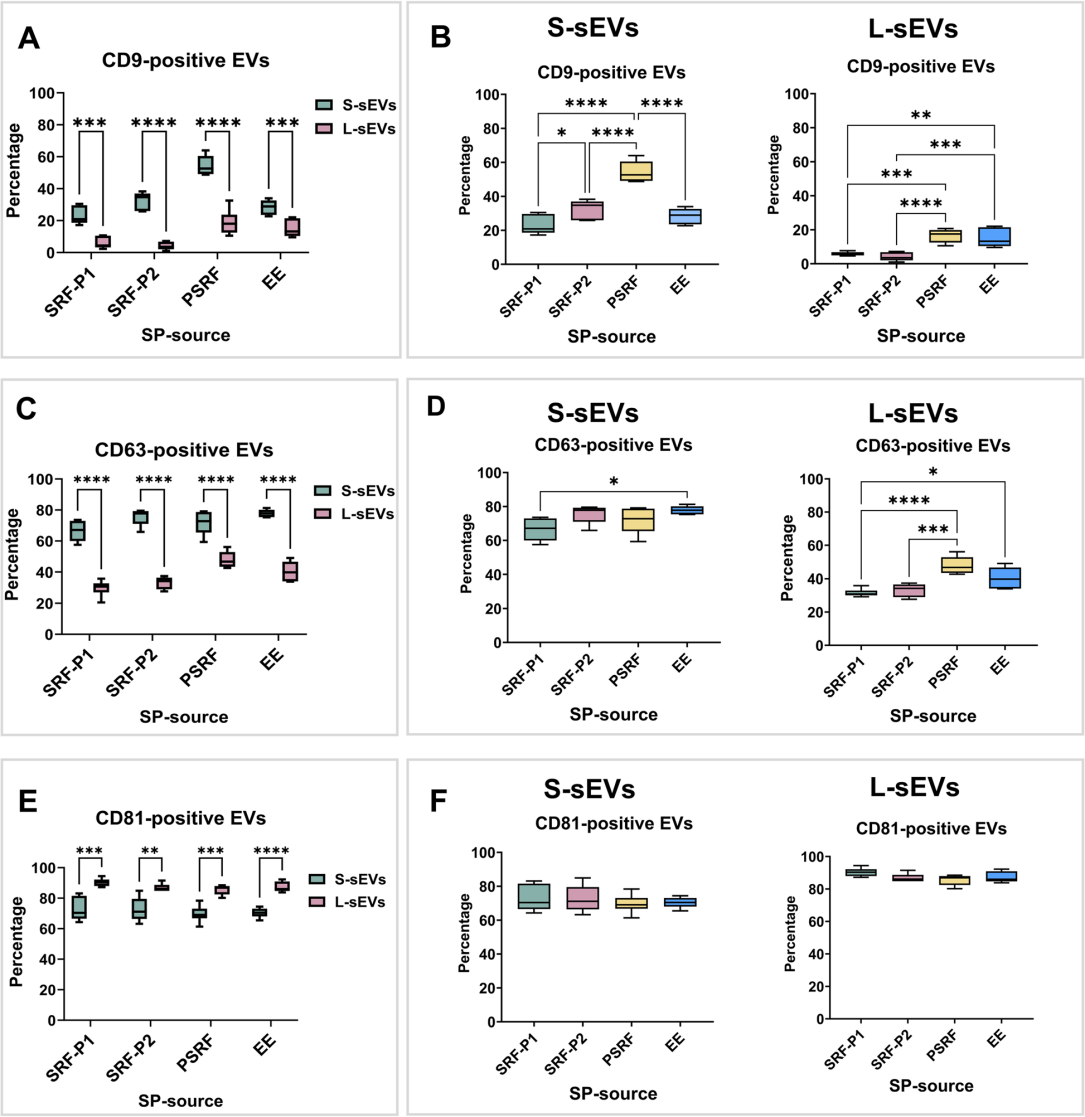
Box plots showing the percentages of porcine seminal plasma (SP) extracellular vesicles (sEVs) expressing the tetraspanins CD9 (**A**-**B**), CD63 (**C**-**D**) and CD81 (**E**-**F**). Seminal EVs were isolated by SEC in two different size subsets (small [S-sEVs] and large [L-sEVs]) and from four different SP sources: the first 10 mL of the sperm rich ejaculate fraction (SRF-P1), the remaining of the SRF (SRF-P2), the post-SRF (PSRF), and from the entire ejaculate (EE). Data from six replicates. Boxes enclose the 25^th^ and 75^th^ percentiles, whiskers extend to the 5^th^ and 95^th^ percentiles, and the line indicates the median. Different asterisks indicate different statistical values (*: *P < *0.05; **: *P <* 0.01; ***: *P <* 0.001; ****: *P <* 0.0001)

Regarding tetraspanins expression (Fig. 3A-B), the percentage of CD9-positive sEVs ranged from 1.01 to 63.97% and differed between the two sEV subsets (*P* < 0.0001) and between the four SP sources (P < 0.0001), with a significant interaction (*P <* 0.0001) between them. The percentage of CD9-positive sEVs was higher in S-sEV (ranging from 17.24 to 63.97%) than in L-sEV (ranging from 1.01 to 32.51%) samples in all SP sources (Fig. 3A). In the S-sEVs, the highest percentage of CD9-positive sEVs was in the PSRF samples, while in the L-sEVs, it was found in PSRF and EE samples (Fig. 3B). The percentage of CD63-positive sEVs ranged from 20.52 to 81.27% and differed between the two sEV subsets (*P <* 0.0001) and between the four SP sources (*P <* 0.0001), with a significant interaction (*P <* 0.01) between them (Fig. 3C, D). The percentage of CD63-positive sEVs was higher in S-sEVs (ranging from 57.51 to 81.27%) than in L-sEVs (ranging from 20.52 to 56.06%) across all SP sources (Fig. 3C). For S-sEVs, the percentage differed between SRF-P1 and EE samples, which had the lowest and highest percentages, respectively. For L-sEVs, the percentage of CD63 positive sEVs was lowest in SRF-P1 and SRF-P2 samples (Fig. 3D). The percentage of CD81 positive EVs ranged from 61.34 to 94.42% and differed between the two sEV subsets (*P < *0.0001) but not between the SP sources, with no significant interaction between them (Fig. 3E, F). The percentages were lower in S-sEVs (ranging from 61.34 to 84.90%) than in L-sEVs (ranging from 83.18 to 94.42%) (Fig. 3E).

**Fig. 4:**
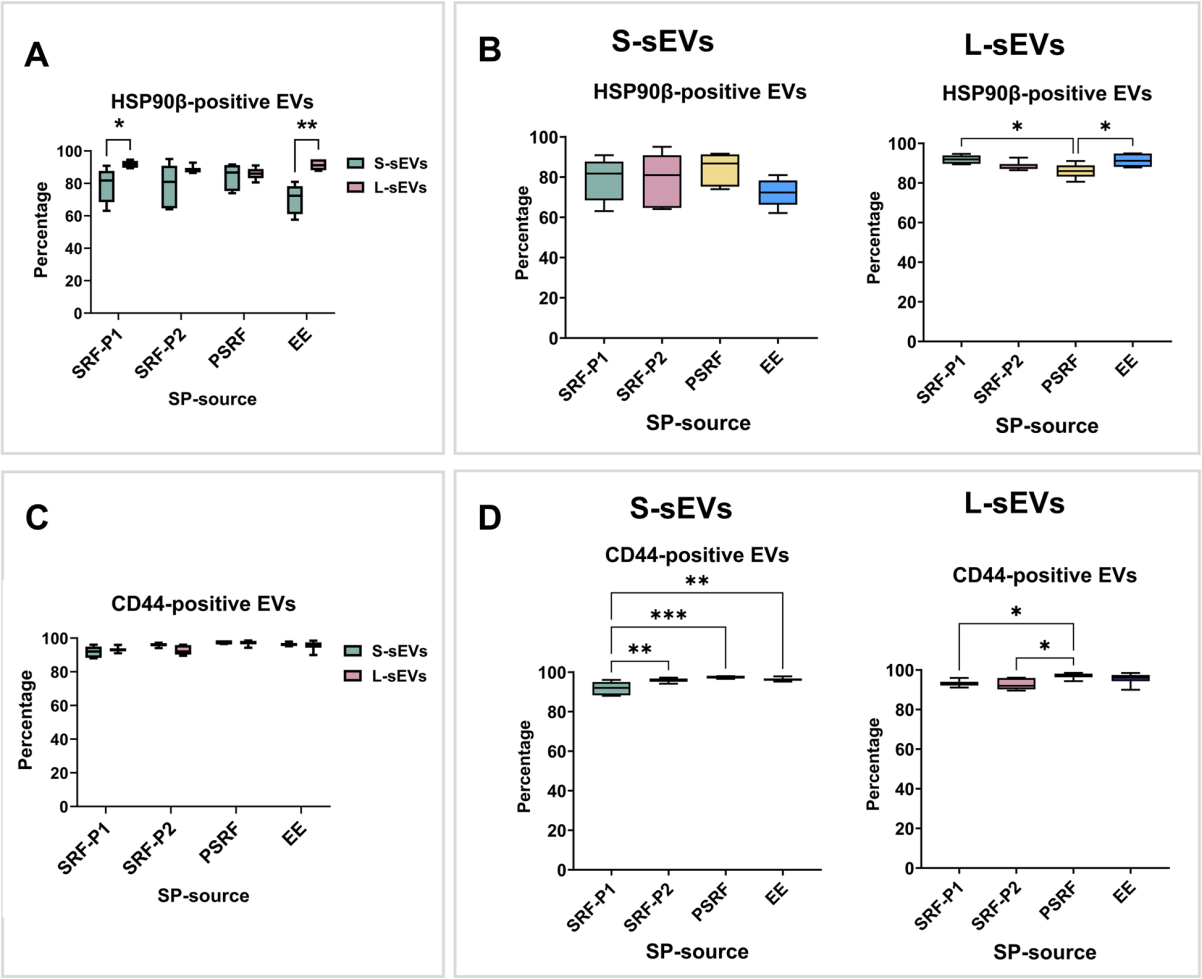
Box plots showing the percentages of porcine seminal plasma (SP) extracellular vesicles (sEVs) expressing HSP90β (**A**-**B**) and CD44 (**C**-**D**). Seminal EVs were isolated by SEC in two different size subsets (small [S-sEVs] and large [L-sEVs]) and from four different SP sources: the first 10 mL of the sperm rich ejaculate fraction (SRF-P1), the remaining of the SRF (SRF-P2), the post-SRF (PSRF), and from the entire ejaculate (EE). Data from six replicates. Boxes enclose the 25^th^ and 75^th^ percentiles, whiskers extend to the 5^th^ and 95^th^ percentiles, and the line indicates the median. Different asterisks indicate different statistical values (*: *P <* 0.05; **: *P < *0.01; ***: *P <* 0.001; ****: *P <* 0.0001)

Regarding the expression of the cytosolic protein HSP90β, the percentage of positive sEVs ranged from 57.66 to 95.03% and differed between the two sEV subsets (*P < *0.0001) but not between the SP sources, with a significant interaction (*P* < 0.05) between them. The percentages were lower (*P <* 0.05) in S-sEVs (ranging from 57.66 to 95.03%) than in L-sEVs (ranging from 80.59 to 94.85%) for SRF-P1 and EE samples (Fig. 4A). Regarding the SP sources in each sEV subset, there were no differences in the S-sEVs, with high percentages of HSP90β-positive sEVs in all SP sources (Fig. 4A). In contrast, there were differences (*P <* 0.05) between SP sources in the L-sEVs, with lower percentages of HSP90β-positive sEVs in the PSRF samples than in the SRF-P1 and EE samples (Fig. 4B).

For the transmembrane protein CD44, the percentage of positive sEVs ranged from 88.04 to 98.50% and differed between the four SP sources (*P < *0.0001) but not between the two sEV subsets, with no significant interaction between them (Fig. 4C, D). For S-sEVs, the percentage was lowest in SRF-P1, and for L-sEVs, the percentage was lowest in the SRF-P1 and SRF-P2 (Fig. 4D).

**Fig. 5:**
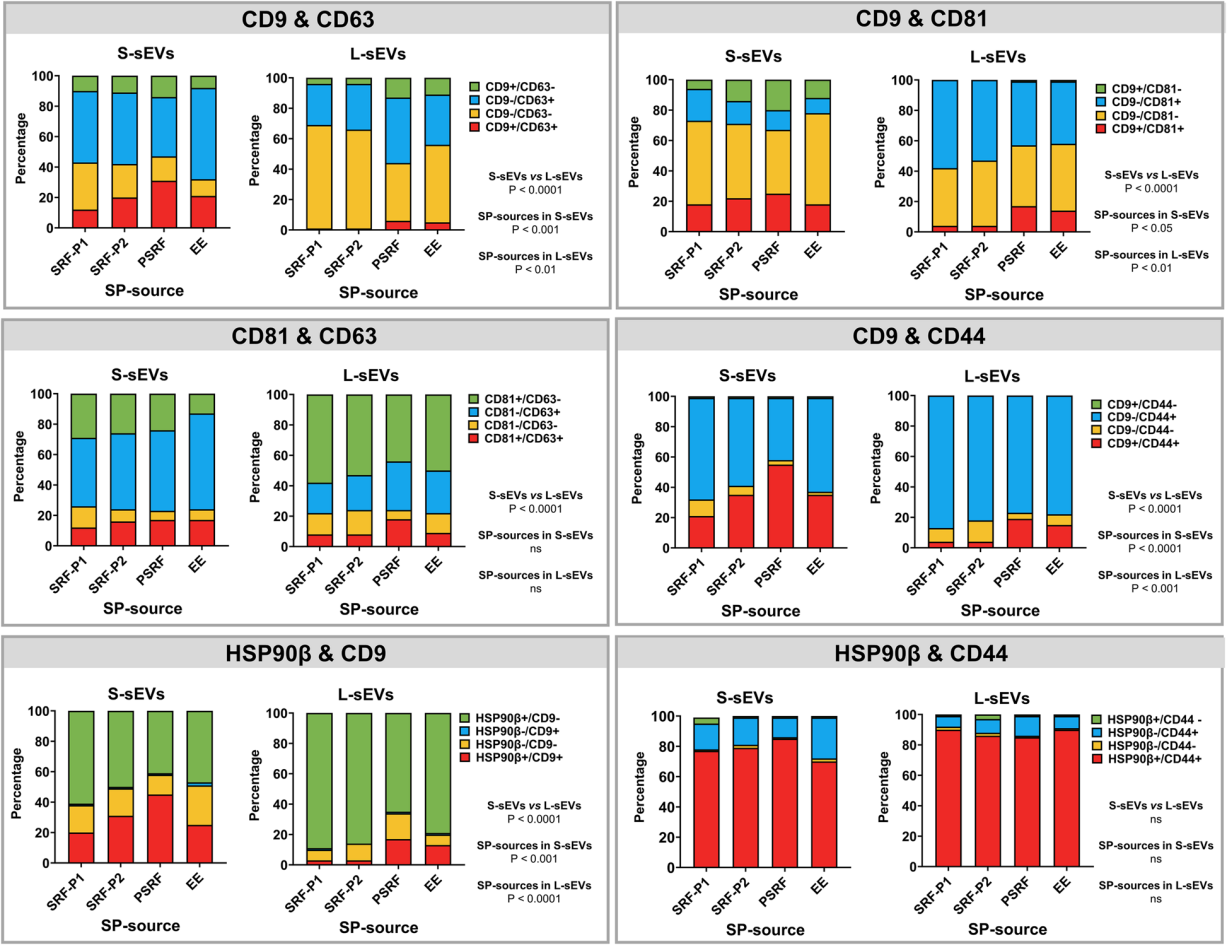
Stacked bar graphs showing the proportion of porcine seminal extracellular vesicles (EVs) simultaneously co-expressing two EV protein markers. Seminal EVs were isolated by SEC in two different size subsets (small [S-sEVs] and large [L-sEVs]) and from four different SP sources: the first 10 mL of the sperm rich ejaculate fraction (SRF-P1), the remaining of the SRF (SRF-P2), the post-SRF (PSRF), and from the entire ejaculate (EE). Bars show data from six replicates. ns indicates no significant differences

Co-expression of CD9/CD63, CD9/CD81, CD81/CD63, CD9/CD44, HSP90β/CD9 and HSP90β/CD44 was analyzed in sEV samples (Fig. 5). Differences (*P* < 0.0001) between sEV subsets (S-sEVs vs L-sEVs) were observed for all of the above antibody combinations except for the HSP90β/CD44 combination, which was expressed by virtually all S-sEVs and L-sEVs. Interestingly, the proportion of sEVs expressing CD9 in combination with any of the other EV protein marker antibodies (CD63, CD81, CD44 and HSP90β) was consistently low in both sEV subsets, but more pronounced in L-sEVs. Differences (*P <* 0.05) between SP sources within each sEV subset were also observed in the proportion of sEVs expressing the CD9/CD63, CD9/CD81, CD9/CD44 and HSP90β/CD9 combinations. Seminal EVs from the PSRF source tended to have the highest percentages of expression for all combinations of EV protein marker antibodies in both sEV subsets.

## Discussion

In recent years, sEVs have emerged as a powerful next-generation biomarker platform for male fertility-related disorders [29]. SP contains a large and heterogeneous population of sEVs [24, 30–34]. Such heterogeneity is a major obstacle to discern the precise role of sEVs. Inconsistent results on the functional role of sEVs in reproductive processes suggest compositional and functional differences between the existing sEV subpopulations [16]. Therefore, the identification and characterization of sEV subpopulations is an open question and at the same time an imperative requirement for a better understanding of the role of sEVs in reproductive processes. The results of the present study, which are based on flow cytometry, are relevant findings that can contribute to the filling of this knowledge gap.

Porcine ejaculate is expelled in two main fractions, SRF and PSRF, which differ in the origin of the SP, being delivered mainly from the prostate and seminal vesicles, respectively [35]. In addition, it has also been found that the SP of the SRF-P1 is mainly derived from the epididymis [35]. Accordingly, in this study, sEVs were isolated into two subsets according to their size (L- and S-sEVs) from three different SP sources (SRF-P1, SRF-P2, PSRF). The isolated sEVs subpopulation could have a different (1) biogenesis, as L-sEVs could be mainly ectosomes and S-sEVs mainly exosomes [1], and (2) cellular origin, as they originated from different reproductive organs. In addition, L-sEVs and S-sEVs isolated from EE, representative of the total sEV population present in the ejaculate, were also analyzed.

Characterization of sEV subtypes, performed according to the MISEV 2018 guidelines [25], confirmed the presence of sEVs in the three ejaculate fractions, in contrast to a previous study that identified sEVs in the SRF but not in the PSRF [36]. The key to this disagreement may have been the sEV isolation method used. Bai et al. [36] used the commercially available Exoquick, while we used the SEC. It is well known that different methods of isolation can lead to different measurable results of the sEVs [37]. The characterization also confirmed, as expected, that S- and L-sEVs differed in size, with S-sEVs being smaller than L-sEVs in all three ejaculate fractions, which would support the suitability of the isolation methods used to isolate two distinct sEV subpopulations of different sizes.

The MISEV 2018 guidelines recommend that the identification of EV-specific protein markers be part of the EV characterization package [25]. Such identification was typically performed using the WB. Flow cytometry is currently a powerful alternative tool to WB because it is more sensitive. The albumin analyses performed would be a clear indication of the higher sensitivity of flow cytometry compared to WB. WB did not detect albumin in either S-sEVs or L-sEVs samples, whereas flow cytometry was able to detect low levels of albumin in both subsets of sEVs. In addition to its higher sensitivity, flow cytometry allows the analysis of individual EVs and thus differentiates EV subpopulations according to their expression or non-expression of proteins of interest [38, 39]. However, not all flow cytometers are well suited for this purpose [13]. The nanometer size of EVs and their low refractive index are limiting factors, as they are outside the sensitivity range of most flow cytometers [40]. In fact, most conventional flow cytometers are unable to detect and immunophenotype the vast majority of EVs because the reduced surface area of EVs results in low expression of surface proteins that can be masked by background noise [39, 41]. The Cytoflex S flow cytometer used in this study has been shown to be useful for EV analysis [13, 42–45] and has been successfully used for surface protein identification on porcine sEVs [24, 46–48].

For the analysis of sEVs by flow cytometry, the adaptation of the cytometer for the analysis of nanosized particles and its subsequent validation by experienced cytometrists is essential, otherwise inaccurate results may be obtained [49]. The optical setup of the flow cytometer was modified by switching from conventional to 405 nm laser-derived SSC detection (violet-SSC), as violet-SSC detection improves the resolution of EVs compared to conventional SSC detection [50]. Calibrating the flow cytometer using reference materials is also critical to accurately match flow rate, light scatter, and fluorescence [38]. It also ensures that EV signals are expressed in standardized units, making the data easier to interpret [38]. In the present study, the flow cytometer was calibrated using polystyrene beads of known diameter (between 80 and 300 nm), and the scattering sensitivity was determined by measuring the smallest bead distinguishable from noise, which was 80 nm. The Mie scattering algorithm was used to achieve a more accurate detection of EVs and to avoid any bias, as the refractive index of polystyrene beads is higher than that of EVs [51]. Recombinant exosomes expressing GFP were used as control to validate the sensitivity of the flow cytometer for the analysis of sEVs and to ensure that the defined EV region was correct.

Labeling of EVs to identify them among the population of nanosized particles collected should be the first step in flow cytometry EV analysis. Efficient EV-labeling dyes should be used, and several lipid-, protein- and nucleic acid-binding fluorescent dyes have been proposed, including CFSE, BODIPY, long-chain dialkylcarbocyanines (DiD, Dil and DiO), PKH67, PKH26, among others [27, 52]. In our study, sEVs were labeled using CFSE, a fluorescent membrane-permeable amine-reactive dye that is widely used for EV labeling [27, 53–55], even for those isolated from porcine SP [24, 46–48]. CFSE was chosen because it does not promote aggregate formation and therefore does not alter the typical light-scattering pattern of EVs [27]. The proportion of CFSE-positive events was higher in L- than in S-sEVs, indicating a higher proportion of non-EV particles in S-sEV samples, which would mostly be lipoproteins of similar size to small EVs [56]. The evidence of a higher albumin content in S- than L-SEV samples would also support this hypothesis.

For sEV immunophenotyping, five EV marker proteins were analyzed by flow cytometry, namely the tetraspanins CD9, CD63 and CD81, the cytosolic protein HSP90β and the transmembrane protein CD44. All are EV surface proteins, with the exception of HSP90β. This is a cytosolic protein that has been shown to diffuse to the membrane surface of EVs [57]. The first four proteins are among the EV-specific marker proteins proposed by MISEV2018, and CD44 was chosen because it has been identified in porcine sEVs [28]. The choice of antibodies is a critical decision, as they must highlight proteins that are present at low levels in nano-sized membrane structures [58]. The first obvious selection criterion should be that the antibodies show reactivity against proteins of the species under study, in this case pig. Unfortunately, there are not many commercial antibodies that meet this requirement. The antibodies used in this study against the three tetraspanins did not meet this requirement, as they were reactive with human but not with porcine tetraspanins. However, the similarity and arrangement of amino acids between human and porcine in these three tetraspanins is very high, ensuring the ability of the selected antibodies to correctly bind to porcine tetraspanins. The brightness of the fluorochrome conjugating the antibody is another important feature to consider. Ensuring adequate brightness is a necessary approach to improve the detection threshold of the flow cytometer for phenotyping a small number of proteins or detecting small EVs [39]. Accordingly, the antibodies used in this study were conjugated to fluorophores with appropriate brightness.

Flow cytometric analysis of sEVs revealed a distinct immunophenotypic signature for each of the eight isolated sEV subpopulations. Detection of the tetraspanins CD9, CD63, and CD81 is a common step in the characterization of EVs, including those isolated from SP [46, 59, 60]. Focusing on the two sEV subsets isolated in the present study, more S- than L-sEVs expressed CD63 and CD9 and less S- than L-sEVs expressed CD81. The expression pattern of these tetraspanins in porcine sEVs differed from that reported in our first study [46]. Different methods of EV isolation would probably explain the discrepancy [38]. In the present study L- and S-sEVs were isolated separately by an SEC-based procedure, whereas in the Barranco et al. [46] study sEVs were isolated in bulk by ultracentrifugation and subsequently sorted into small and large by SSC flow cytometry. Ultracentrifugation remains the gold standard for EV isolation. However, SEC better preserves the morphological structure of isolated EVs [61]. Furthermore, the isolation method affects the proteome and transcriptome of the isolated EVs [62, 63]. These observations highlight the urgent need for standardization of sEV isolation methods, selecting those that best preserve the integrity and molecular composition of sEVs.

Focusing on the ejaculate fractions, sEVs expressing CD9 (in both sEV subsets) and CD63 (only in L-sEVs) were more abundant in PSRF than in SRF. Alvarez-Rodriguez et al. [28] found no differences between porcine ejaculate fractions in the proportions of sEVs expressing CD9, CD63, or CD81. Differences in the sEV isolation method (two-step discontinuous density gradient ultracentrifugation *vs* SEC) and the flow cytometry protocol used to identify tetraspanins in sEVs (EXO-FLOW™ exosome purification beads vs direct staining) between the two studies may explain the different results. The differences found in the immunophenotypic sEV profile between PSRF and SRF would reflect a different cellular origin, as EVs express the phenotype of cells of origin [64, 65]. Tetraspanins can characterize the cargo and interaction of EVs with target cells [66]. Therefore, the different profile in tetraspanins among sEVs from different ejaculate fractions could indicate that each subpopulation of sEVs would have different cargo and target cells, in addition to a different cellular origin. Although the role of sEV tetraspanins in male reproductive processes is still unclear, Caballero et al. [67] found in bull epididymosomes that only those expressing CD9 were able to bind and transfer their cargo to live epididymal spermatozoa. These researchers also reported that CD9-expressing epididymosomes were particularly enriched in proteins involved in sperm maturation and sperm-oocyte interaction. A beneficial effect of sEVs expressing CD9 and CD63 on sperm quality and functional parameters was observed in pigs by Du et al. [60].

The protein HSP90β was another EV marker analyzed and it was more expressed in L-sEVs, but only in those isolated from the SRF-P1. The role of HSPs in EVs is still unclear. However, HSP90 proteins would be involved in the fusion of multivesicular body vesicles to the plasma membrane [68]. Ono et al. [57] found that HSP90β was expressed in EVs released from human metastatic oral cancer cells but not from parenteral cells, suggesting that its expression level in EVs may depend on their cellular origin. In male reproduction, HSP90 proteins have been identified in sperm from several mammalian species [69] and have been positively associated with relevant sperm functions, including motility, hyperactivation, and acrosome reaction [70, 71]. Lower sperm HSP90β expression has also been associated with male fertility disorders [71, 72]. It is challenging to investigate whether the differential HSP90β expression between sEV subpopulations could be related to functional changes in spermatozoa or male fertility.

The last protein analyzed in sEVs was CD44. Interestingly, CD44 was expressed by virtually in all sEVs, regardless of sEV subtype or SP source. These results are consistent with those reported by Alvarez-Rodriguez et al. [28], who observed that the majority of porcine sEVs were CD44-positive. This ubiquity may support the use of CD44 as a universal protein marker for the identification of the entire porcine sEV population. However, further studies are needed to confirm this statement. This finding was not particularly surprising, as CD44 has been identified in the more important sEV release organs of the male reproductive tract, such as the epididymis and prostate [73], as well as in spermatozoa [74]. The CD44 protein is the major cell surface receptor for hyaluronan [75] and thus may play a critical role in reproductive events, as hyaluronan is involved in sperm survival and capacitation, as well as successful sperm storage in the oviduct [76]. Whether CD44 of sEVs could be involved in these functional male reproductive events would be an interesting research topic for clarification.

Although flow cytometry is a valuable technology for the analysis of EVs, it has some drawbacks, mainly due to the limitation of the instrument to detect and analyze small EVs (diameter < 100 nm), which have a small surface area with low protein expression and low scattering properties [39]. This is of utmost importance considering that EV size ranges from 30 to 1000 nm and is particularly relevant for exosomes (small EVs), whose size ranges from 30 to 150 nm [1]. Indeed, one of the weaknesses of the present study was that the flow cytometer was unable to detect EVs smaller than 118 nm. As result, the smaller sEVs, which were mainly present in the S-sEV samples as shown by DLS and TEM, were neither detected nor analyzed. This instrumental limitation was the reason why the concentration of sEVs was not measured by flow cytometry. Another weakness of the present study was that fluorescence calibration was not performed, which would certainly have been beneficial for follow-up studies [22].

The characterization of single EVs is becoming increasingly important both for the phenotyping of EVs and for unraveling the key roles of the EVs in physiological/pathological processes. In recent years, advances in innovative technologies for the analysis of single EVs have made possible to overcome the limitations of EV characterization and phenotyping due to their highly heterogeneous nature and nanometric size. In addition to high sensitive flow cytometers (including Cytoflex S, Apogee or NanoFCM), other technologies using label (nanoparticle coating or fluorescence) or label-free techniques for EV analysis have been described [10, 77, 78]. Some examples of label-free technologies include cryogenic electron microscopy, atomic force microscopy, Raman tweezer micro spectroscopy, or single-particle interferometric reflectance imaging sensor. Label-based technologies include total internal reflection fluorescence microscopy, resonance energy transfer, super-resolution microscopy, or digital droplet PCR. However, each of these technologies has specific strengths and weaknesses, and still needs to improve its detection capability, cost, and performance [77].

## Conclusions

In conclusion, this experimental study demonstrates the suitability of high-sensitivity flow cytometry for the immunophenotyping of sEVs and for the identification of distinct subpopulations within the heterogeneous population of EVs present in SP, in this case in porcine SP. The identified subpopulations of sEVs would have different cellular origins and may have different cargoes, functions, and target cells. Notably, the flow cytometry analysis performed did not identify the same proportion of L- and S-sEVs. While it was able to identify almost all L-sEVs, it did not identify the same proportion of S-sEVs, as those smaller than 118 nm were not identified. Current high-sensitivity flow cytometers, such as the Cytoflex S used here, are unable to detect EVs smaller than 100 nm, making it impractical to analyze and phenotype the full range of EV sizes. This fact, which is undoubtedly a limitation of the present study, does not diminish the value of the results obtained, but should be considered in the use of these results in future studies.

### Supplementary Information

**Additional file 1.** MIFlowCyt-EV report. Detailed description of the flow cytometry analysis, including pre-analytical and analytical procedures.

**Additional file 2.** MIFlowCyt report. Detailed description of the flow cytometry analysis, including pre-analytical and analytical procedures.

**Additional file 3:** **Supplementary Fig. 1.** Characterization of the quantity (indirectly assessed by total protein concentration) and size distribution (dynamic light scattering analysis; DLS) of seminal extracellular vesicles (sEVs) isolated using a size exclusion chromatography-based method from porcine ejaculates. Eight subpopulations of sEVs were generated based on two size sEV subsets (small [S-sEVs] and large [L-sEVs]) from four different seminal plasma (SP) sources: the first 10 mL of the sperm rich ejaculate fraction (SRF-P1), the remaining of the SRF (SRF-P2), the post-SRF (PSRF) and entire ejaculate (EE). (A) Violin plot showing total protein concentration (μg/mL). The dashed lines indicate the median and the dotted lines indicate the interquartile range from 25 to 75%. The table below the graph shows total protein concentration data (mean ± SD) for each sEV subset and SP source. (B) Particle size distribution (nm) in each sEV sample. Solid and dashed lines represent L-sEVs and S-sEVs, respectively. Each color represents one SP source. The table below the graph shows the size distribution data (mean ± SD) for each sEV subset and SP source. (C) Violin plot showing Zeta potential (mV). The dashed lines indicate the median and the dotted lines indicate the interquartile range from 25 to 75%. Each color represents one SP source. Data are from six biological replicates, each containing an SP pool of five ejaculates.

**Additional file 4:** **Supplementary Fig. 2.** Representative transmission electron microscopy images of extracellular vesicles isolated from porcine seminal plasma (sEVs). Eight subpopulations of sEVs were generated based on two size sEV subsets (small [S-sEVs] and large [L-sEVs]) from four different seminal plasma (SP) sources: the first 10 mL of the sperm rich ejaculate fraction (SRF-P1), the remaining of the SRF (SRF-P2), the post-SRF (PSRF) and entire ejaculate (EE).

**Additional file 5:** **Supplementary Fig. 3.** (A) Representative image (cropped) of Western blot (WB) analysis of albumin in porcine seminal plasma (SP, positive control) and two extracellular vesicle (sEV) size subsets (small [S-sEVs] and large [L-sEVs]) isolated from porcine seminal plasma using a size exclusion chromatography-based method; (B) Full scan (uncropped) of WB image.

**Additional file 6:** **Supplementary Fig. 4.** Determination of Cytoflex S sensitivity and extracellular vesicle (EV) forward scatter (FSC) and side scatter (violet-SSC, vSSC) region for analysis of porcine seminal EVs. (A) Commercially available recombinant exosomes expressing green fluorescent protein (GFP) on their membrane surface (SAE0193, Merck^®^) were used and the region of interest was defined based on their FSC/vSSC characteristics, gating the area where EVs used as standards occur. The GFP signal was used as a threshold. (B) Based on this previous analysis, CFSE-stained sEVs were used as a control for the sEVs preparation, using the region defined by the standards and using FSC/vSSC as a threshold. Immunophenotyping analysis was not performed if the percentage of CFSE-positive events was less than 50%. CFSE: carboxyfluorescein succinimidyl ester.

**Additional file 7:** **Supplementary Fig. 5.** Flow cytometry controls performed to characterize and immunophenotype porcine seminal extracellular vesicles (sEVs). Representative time vs violet side scatter (V-SSC-A) dot plots of (A) a sample of 0.1 μm filtered phosphate buffer saline (PBS), and (B) a sample of 0.1 μm PBS with CFSE and each antibody tested. (C) Representative dot plots of unstained sEV samples. Representative forward scatter (FSC-H) vs violet side scatter (V-SSC-H) dot plots of (D) a sample of 0.1 μm PBS, (E) a sample of 0.1 μm PBS with CFSE, (F) with antibodies, and (G) with unstained sEV samples. Note the low number of events in A and B, and the complete absence of fluorescence-positive events in C.

**Additional file 8:** **Supplementary Fig. 6.** Flow cytometry calibration controls performed to characterize and immunophenotype small (S-) and large (L-) porcine seminal extracellular vesicles (sEVs). Representative plot (violet side scatter [violet-SSC]/forward side scatter [FSC]) for each antibody showing the number of events falling within the sEV region.

**Additional file 9:** **Supplementary Fig. 7.** Flow cytometry controls performed to characterize and immunophenotype porcine seminal extracellular vesicles (sEVs). Representative dot plots of non-lysed (left) and lysed (right) samples of porcine seminal extracellular vesicles (sEVs) stained with CD44-FITC + HSP90β-PE (top) and CD81-APC + CD44-FITC (bottom). The lysis detergent solution was Triton (0.1%) and sodium dodecyl sulfate (0.1%). Note the absence of positive events in the lysed sEV samples.

## Data Availability

The datasets used and analyzed during the current study are available from the corresponding author on reasonable request. The datasets generated during the current study are available in the FlowRepository database (http://flowrepository.org/id/FR-FCM-Z732).

## Abbreviations

AI: Artificial insemination
APC: Allophycocyanin
CFSE: Carboxyfluorescein succinimidyl ester
DLS: Dynamic light scattering
DMSO: Dimethyl sulfoxide
EE: Entire ejaculate
EVs: Extracellular vesicles
FITC: Fluorescein isothiocyanate
FSC: Forward scatter
GFP: Green fluorescent protein
HSP: Heat shock protein
L-sEVs: Large seminal extracellular vesicles
MISEV: Minimal information for studies of extracellular vesicles
PBS: Phosphate buffered saline
PE: Phycoerythrin
PerCP: Peridinin Chlorophyll Protein
PSRF: Post sperm rich ejaculate fraction
RT: Room temperature
S-sEVs: Small seminal extracellular vesicles
SDS: Sodium dodecyl sulfate
SEC: Size exclusion chromatography
sEVs: Seminal extracellular vesicles
SP: Seminal plasma
SRF-P1: First 10 mL of the sperm rich ejaculate fraction
SRF-P2: Remaining sperm rich ejaculate fraction
SSC: Side scatter
TBS: Tris-buffered saline
TEM: Transmission electron microscopy
vSSC: Violet side scatter
WB: Western blot
