# Supplementary material for: Immunophenotype profile by flow cytometry reveals different subtypes of extracellular vesicles in porcine seminal plasma
Source: Cell Commun Signal. 2024 Jan 23;22:63. doi: 10.1186/s12964-024-01485-1 (PMC10807091; doi:10.1186/s12964-024-01485-1)
Supplement: Supplementary file 2 — Additional file 2. MIFlowCyt report. Detailed description of the flow cytometry analysis, including pre-analytical and analytical procedures. [file 12964_2024_1485_MOESM2_ESM.doc]

| **Requirement** | **Please Include Requested Information** |
| --- | --- |
| 1.1. Purpose | Immunophenotyping of porcine seminal plasma extracellular vesicles (sEVs) subpopulations by high-resolution flow cytometry |
| 1.2. Keywords | Ejaculate, extracellular vesicles, flow cytometry, pig, seminal plasma. |
| 1.3. Experiment variables | Not applicable. |
| 1.4. Organization name and address | 1. Department of Medicine and Animal Surgery, Faculty of Veterinary Science, University of Murcia, Spain.  2. Servicio de Técnicas Aplicadas a las Biociencias, Universidad de Extremadura, Badajoz, Spain. |
| 1.5. Primary contact name and email address | Isabel barranco, Isabel.barranco@um.es |
| 1.6. Date or time period of experiment | 2022-2023 |
| 1.7. Conclusions | This experimental study demonstrates the suitability of high-resolution flow cytometry for the immunophenotyping of extracellular vesicles (EVs) and thus for the identification of different subpopulations within a population of EVs, in this case that of porcine seminal plasma. The identified subpopulations of sEVs would have different cellular sources and may have different cargoes, functions, and target cells. Notably, the flow cytometry analysis performed did not identify the same proportion of large (L-sEVs) and small (S-sEVs). While it was able to identify almost all L-sEVs, it did not identify the same proportion of S-sEVs, as those smaller than 118 nm were not identified. Current high-resolution flow cytometers, such as the Cytoflex S used here, are unable to detect EVs smaller than 100 nm, making it impractical to analyze all EVs. This fact, which is undoubtedly a limitation of the present study, does not affect the merit of the results obtained, but should be considered when using them in future studies. |
| 1.8. Quality control measures | Revise MIFlowCyt_EV file |
| 2.1.1.1. (2.1.2.1., 2.1.3.1.) Sample description | Revise MIFlowCyt_EV file |
| 2.1.1.2. Biological sample source description | Revise MIFlowCyt_EV file |
| 2.1.1.3. Biological sample source organism description | Revise MIFlowCyt_EV file |
| 2.1.2.2. Environmental sample location | Revise MIFlowCyt_EV file |
| 2.3. Sample treatment description | No treatment was applied |
| 2.4. Fluorescence reagent(s) description | CellTrace ™ carboxyfluorescein succinimidyl ester (CFSE, Thermo Fisher Scientific, Waltham, Massachusetts, USA)  Anti-CD9-PerCP (130-118-814, Miltenyi Biotec, Bergisch Gladbach, Germany)  Anti-CD63-FITC (130-123-673, Miltenyi Biotec, Bergisch Gladbach, Germany)  Anti-CD81-APC (130-119-787, Miltenyi Biotec, Bergisch Gladbach, Germany)  Anti-HSP90β-PE (ADI-SPA-844PE, Enzo life Sciences, Sant Cugat del Vallès, Barcelona, Spain)  Anti-CD44-FITC (MCA4703F, Bio-Rad, Hercules, California, USA)  Anti-albumin-FITC (CLFAG16140, Cedarlane Laboratories, Burlington, Canada)  Exosome standard-GFP (SAE0193, Merck, Darmstadt, Germany) |
| 3.1. Instrument manufacturer | Beckman Coulter |
| 3.2. Instrument model | Cytoflex S |
| 3.3. Instrument configuration and settings | The flow cytometer was equipped with four lasers, namely violet, blue, yellow, and red (405 nm, 488 nm, 561 nm and 638 nm, respectively), to detect up to 13 fluorescence parameters. The optical setup of the flow cytometer was modified to use the side scatter (SSC) information of the 405 nm laser (violet-SSC-A) instead of the 488 nm laser. |
| 4.1. List-mode data files | *We recommend all authors to submit their data files to [http://flowrepository.org](http://flowrepository.org/) and to make them available for the peer-review process. If you have done so, please let us know by inserting the following codes (replace the red text):  The files have been submitted to [http://flowrepository.org](http://flowrepository.org/);  1) The link for peer-review process:  http://flowrepository.org/id/RvFr8GMJP6qZ0NnEKxPY4aozcheB7P32804KfhqtSdQT006SzzGljvGznm08ocXT 2) The repository identifier:  http://flowrepository.org/id/FR-FCM-Z732. This link will be made publicly accessible after the paper is published. |
| 4.2. Compensation description | No compensation was needed. PE was excited with 561 nm laser and fluorescence was collected in channel Y1 (575/25 nm filter), FITC was excited with 488 nm laser and fluorescence was collected in channel B1 (525/25-nm filter), PerCP-A was excited with 488 nm laser and fluorescence was collected in channel B2 (710/30 nm filter), and APC was excited with 633 nm laser and fluorescence was collected in channel R1 (670/40 nm filter). |
| 4.3. Data transformation details | Not applicable |
| 4.4.1. Gate description | vSSC/FSC gate |
| 4.4.2. Gate statistics | Higher than 50% |
| 4.4.3. Gate boundaries | Polygonal gate, described in manuscript supplementary figures.  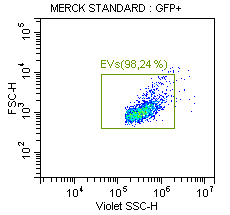 |

**OtNotes**

Feel free to use more space than allocated.

You can embed graphics/figures in this document, if needed.
