# Supplementary figures and images for: Immunophenotype profile by flow cytometry reveals different subtypes of extracellular vesicles in porcine seminal plasma
Source: Cell Commun Signal. 2024 Jan 23;22:63. doi: 10.1186/s12964-024-01485-1 (PMC10807091; doi:10.1186/s12964-024-01485-1)

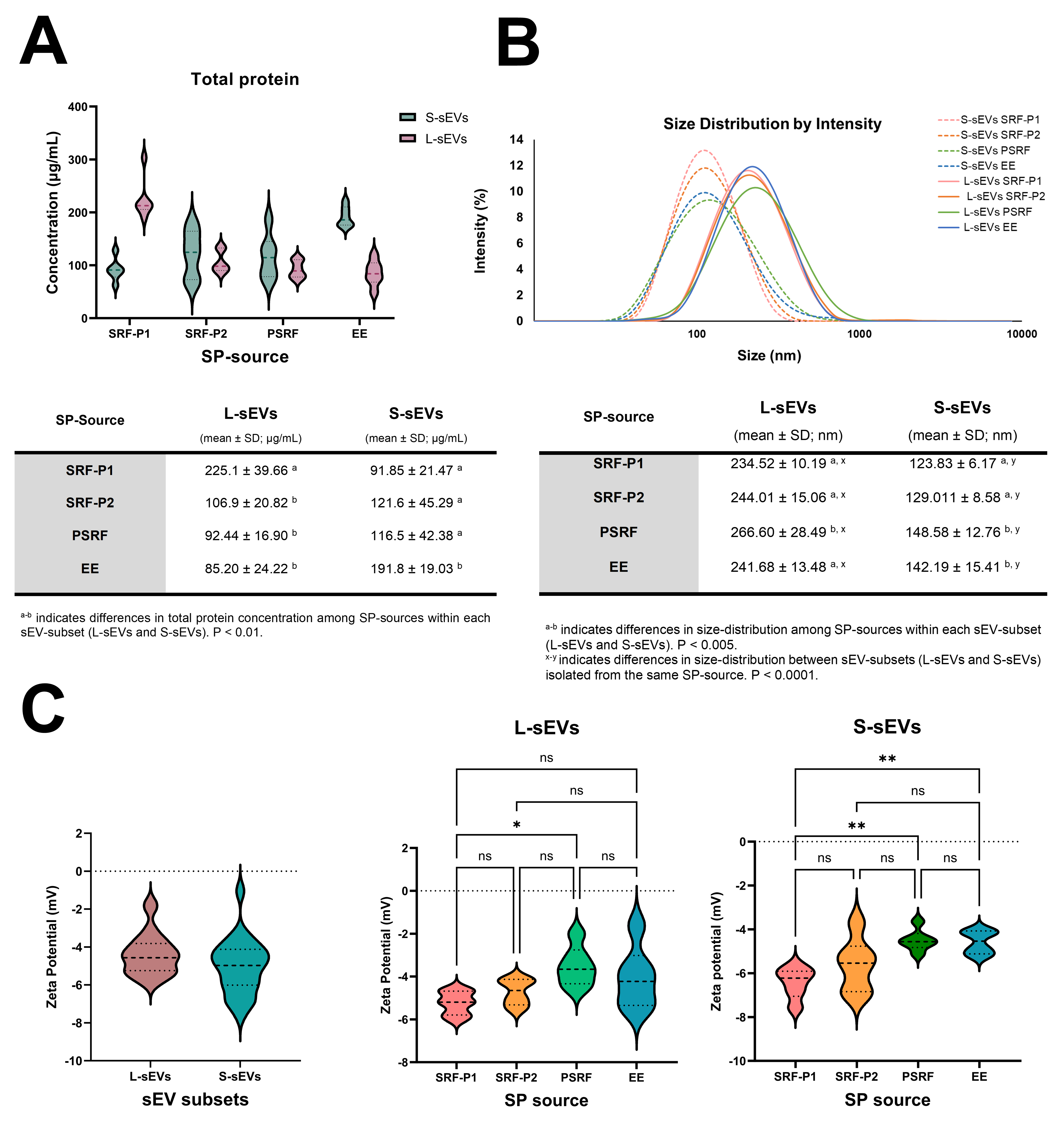

Supplement: Supplementary file 3 — Additional file 3: Supplementary Fig. 1. Characterization of the quantity (indirectly assessed by total protein concentration) and size distribution (dynamic light scattering analysis; DLS) of seminal extracellular vesicles (sEVs) isolated using a size exclusion chromatography-based method from porcine ejaculates. Eight subpopulations of sEVs were generated based on two size sEV subsets (small [S-sEVs] and large [L-sEVs]) from four different seminal plasma (SP) sources: the first 10 mL of the sperm rich ejaculate fraction (SRF-P1), the remaining of the SRF (SRF-P2), the post-SRF (PSRF) and entire ejaculate (EE). (A) Violin plot showing total protein concentration (μg/mL). The dashed lines indicate the median and the dotted lines indicate the interquartile range from 25 to 75%. The table below the graph shows total protein concentration data (mean ± SD) for each sEV subset and SP source. (B) Particle size distribution (nm) in each sEV sample. Solid and dashed lines represent L-sEVs and S-sEVs, respectively. Each color represents one SP source. The table below the graph shows the size distribution data (mean ± SD) for each sEV subset and SP source. (C) Violin plot showing Zeta potential (mV). The dashed lines indicate the median and the dotted lines indicate the interquartile range from 25 to 75%. Each color represents one SP source. Data are from six biological replicates, each containing an SP pool of five ejaculates. [file 12964_2024_1485_MOESM3_ESM.tif]

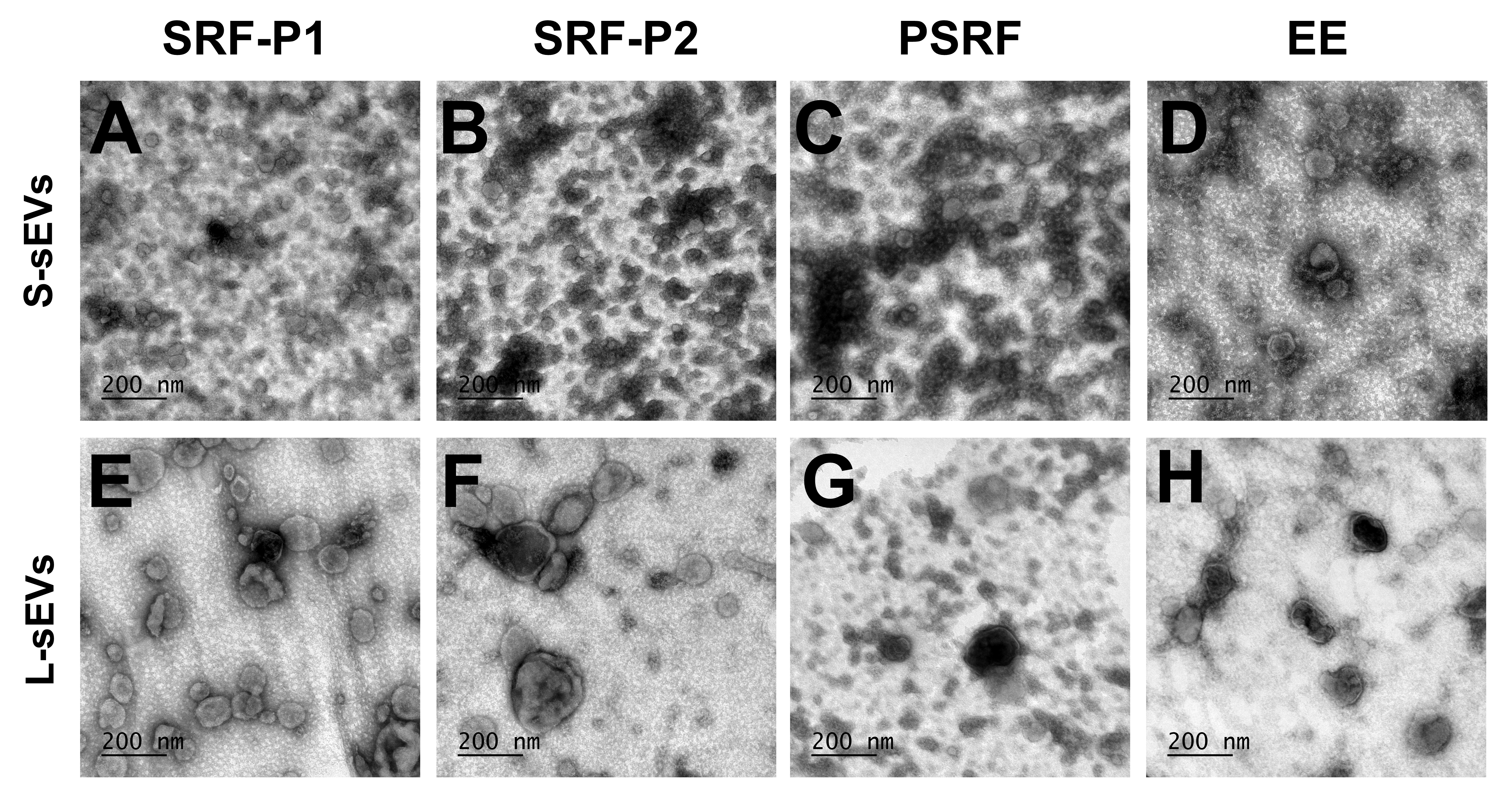

Supplement: Supplementary file 4 — Additional file 4: Supplementary Fig. 2. Representative transmission electron microscopy images of extracellular vesicles isolated from porcine seminal plasma (sEVs). Eight subpopulations of sEVs were generated based on two size sEV subsets (small [S-sEVs] and large [L-sEVs]) from four different seminal plasma (SP) sources: the first 10 mL of the sperm rich ejaculate fraction (SRF-P1), the remaining of the SRF (SRF-P2), the post-SRF (PSRF) and entire ejaculate (EE). [file 12964_2024_1485_MOESM4_ESM.tif]

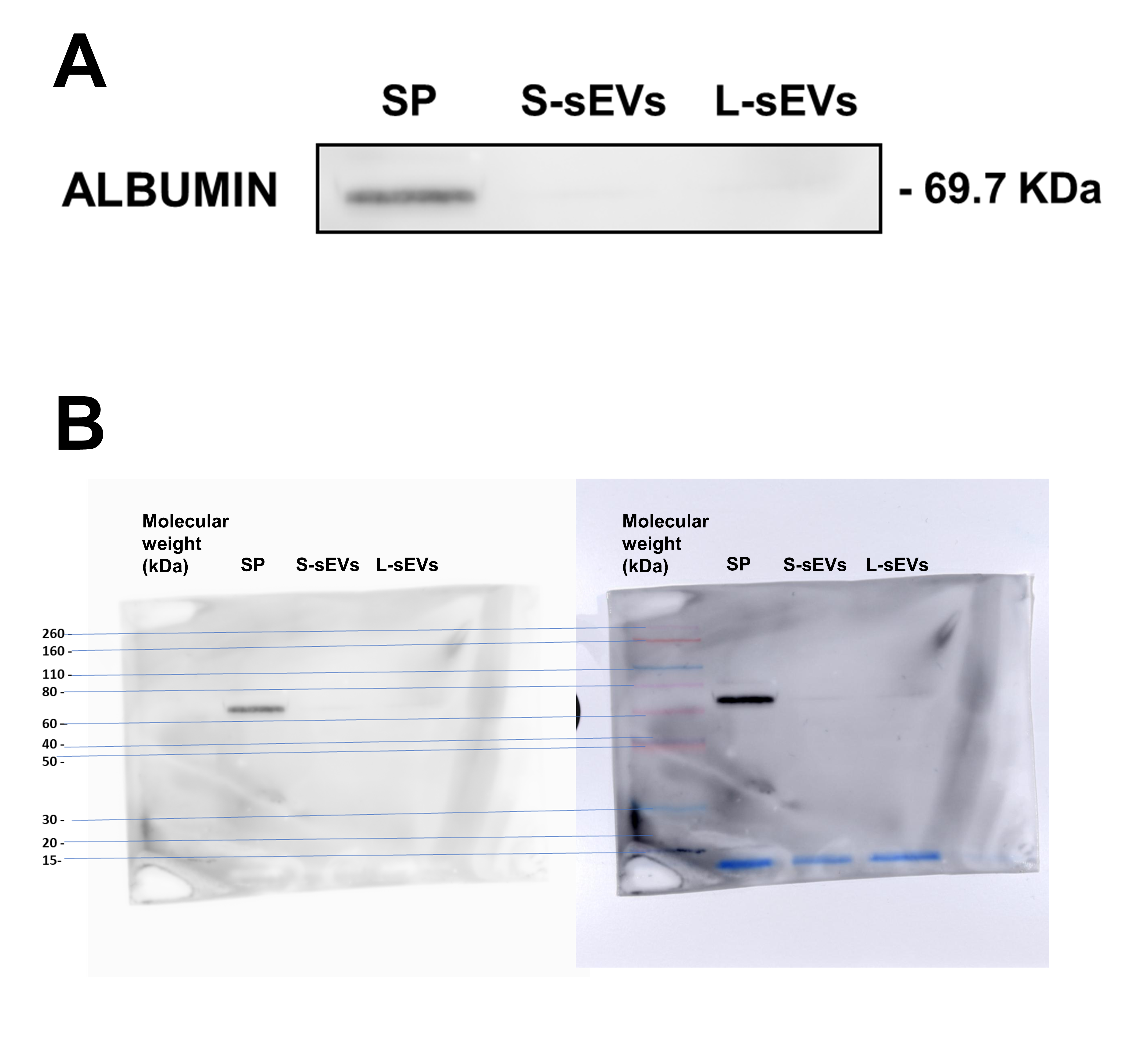

Supplement: Supplementary file 5 — Additional file 5: Supplementary Fig. 3. (A) Representative image (cropped) of Western blot (WB) analysis of albumin in porcine seminal plasma (SP, positive control) and two extracellular vesicle (sEV) size subsets (small [S-sEVs] and large [L-sEVs]) isolated from porcine seminal plasma using a size exclusion chromatography-based method; (B) Full scan (uncropped) of WB image. [file 12964_2024_1485_MOESM5_ESM.tif]

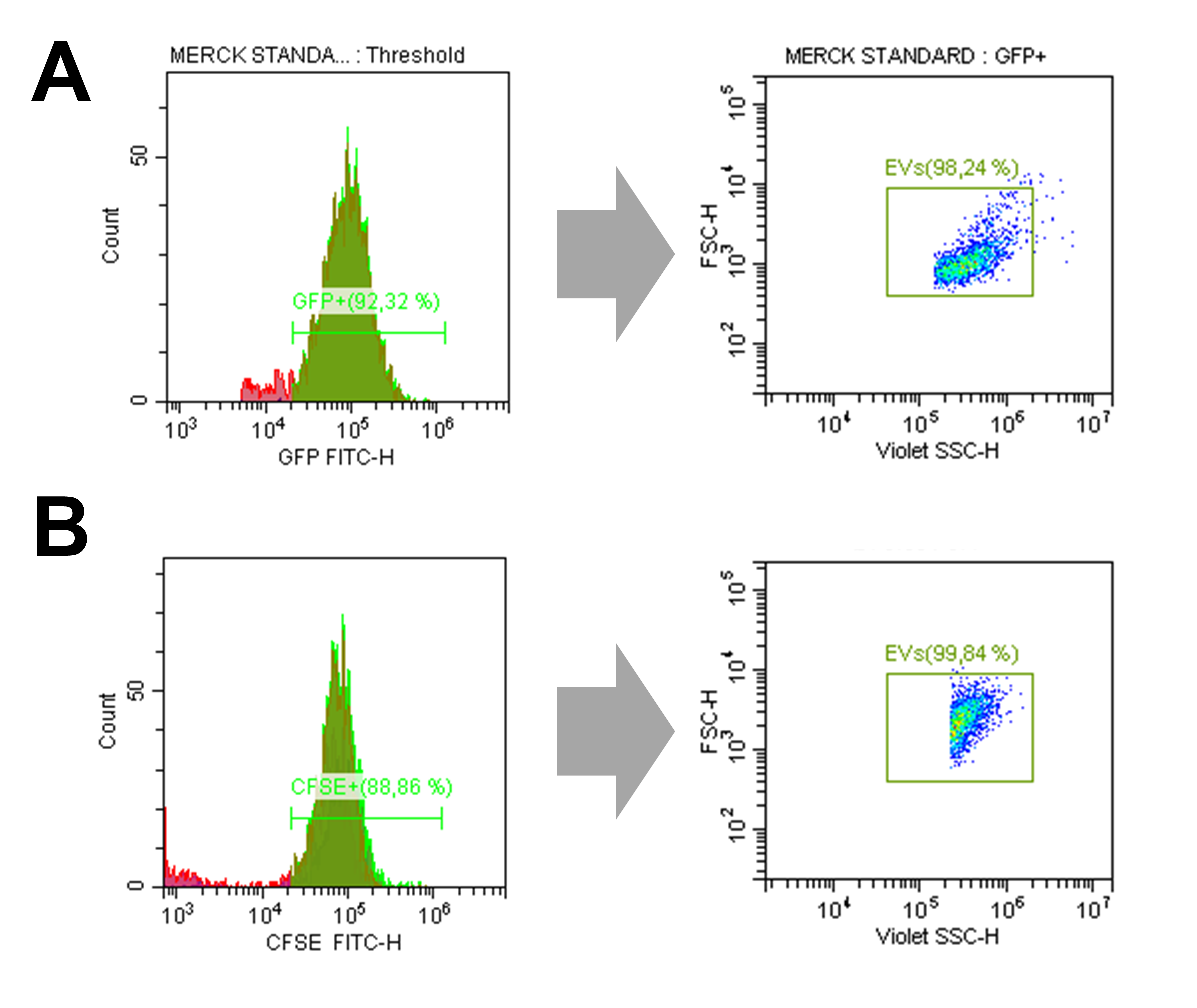

Supplement: Supplementary file 6 — Additional file 6: Supplementary Fig. 4. Determination of Cytoflex S sensitivity and extracellular vesicle (EV) forward scatter (FSC) and side scatter (violet-SSC, vSSC) region for analysis of porcine seminal EVs. (A) Commercially available recombinant exosomes expressing green fluorescent protein (GFP) on their membrane surface (SAE0193, Merck®) were used and the region of interest was defined based on their FSC/vSSC characteristics, gating the area where EVs used as standards occur. The GFP signal was used as a threshold. (B) Based on this previous analysis, CFSE-stained sEVs were used as a control for the sEVs preparation, using the region defined by the standards and using FSC/vSSC as a threshold. Immunophenotyping analysis was not performed if the percentage of CFSE-positive events was less than 50%. CFSE: carboxyfluorescein succinimidyl ester. [file 12964_2024_1485_MOESM6_ESM.tif]

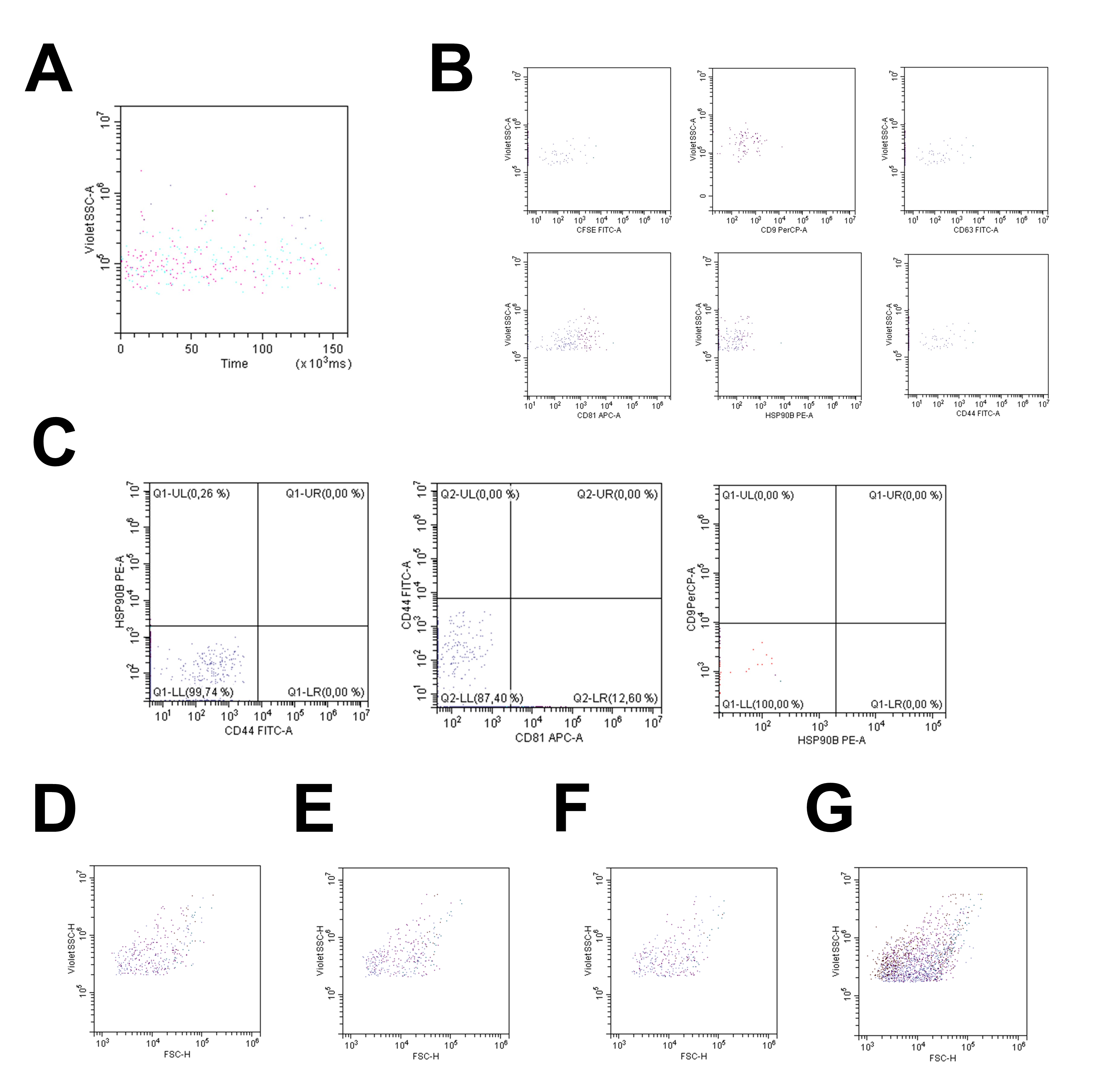

Supplement: Supplementary file 7 — Additional file 7: Supplementary Fig. 5. Flow cytometry controls performed to characterize and immunophenotype porcine seminal extracellular vesicles (sEVs). Representative time vs violet side scatter (V-SSC-A) dot plots of (A) a sample of 0.1 μm filtered phosphate buffer saline (PBS), and (B) a sample of 0.1 μm PBS with CFSE and each antibody tested. (C) Representative dot plots of unstained sEV samples. Representative forward scatter (FSC-H) vs violet side scatter (V-SSC-H) dot plots of (D) a sample of 0.1 μm PBS, (E) a sample of 0.1 μm PBS with CFSE, (F) with antibodies, and (G) with unstained sEV samples. Note the low number of events in A and B, and the complete absence of fluorescence-positive events in C. [file 12964_2024_1485_MOESM7_ESM.tif]

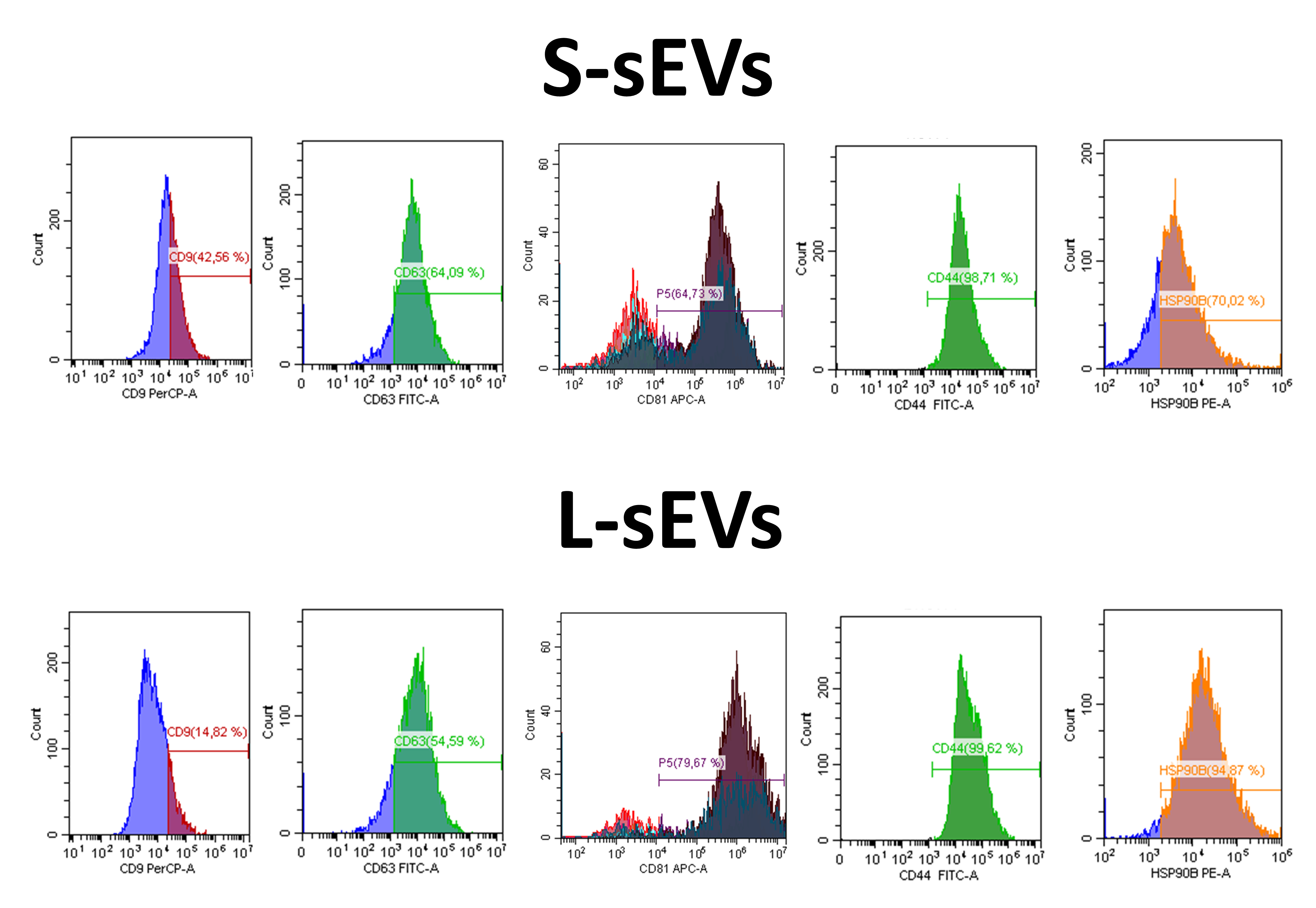

Supplement: Supplementary file 8 — Additional file 8: Supplementary Fig. 6. Flow cytometry calibration controls performed to characterize and immunophenotype small (S-) and large (L-) porcine seminal extracellular vesicles (sEVs). Representative plot (violet side scatter [violet-SSC]/forward side scatter [FSC]) for each antibody showing the number of events falling within the sEV region. [file 12964_2024_1485_MOESM8_ESM.tif]

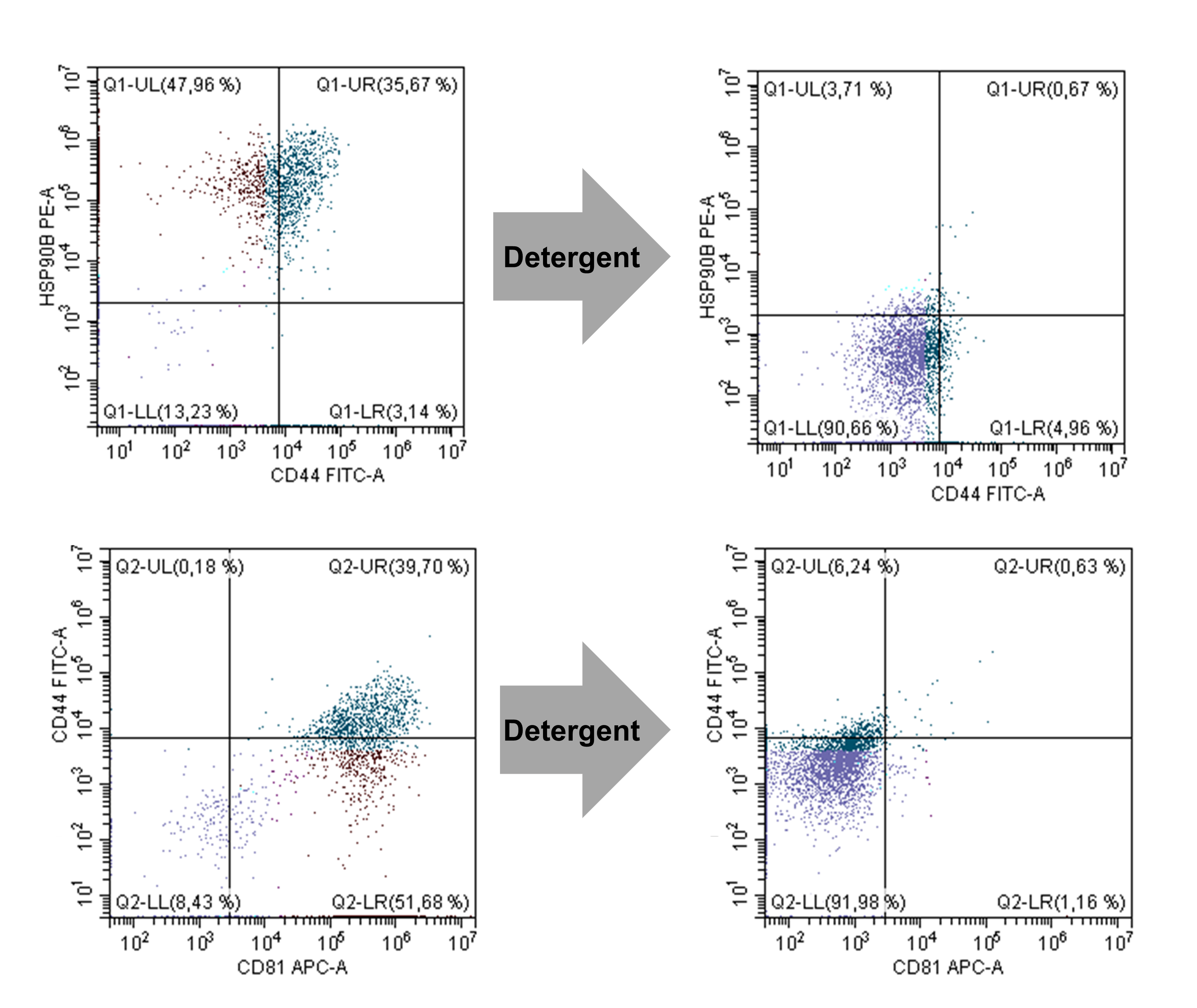

Supplement: Supplementary file 9 — Additional file 9: Supplementary Fig. 7. Flow cytometry controls performed to characterize and immunophenotype porcine seminal extracellular vesicles (sEVs). Representative dot plots of non-lysed (left) and lysed (right) samples of porcine seminal extracellular vesicles (sEVs) stained with CD44-FITC + HSP90β-PE (top) and CD81-APC + CD44-FITC (bottom). The lysis detergent solution was Triton (0.1%) and sodium dodecyl sulfate (0.1%). Note the absence of positive events in the lysed sEV samples. [file 12964_2024_1485_MOESM9_ESM.tif]
